# Educational assessments in entry-level physical therapy education: a scoping review

**DOI:** 10.1186/s12909-026-08927-z

**Published:** 2026-03-05

**Authors:** Rachel S. Tappan, Elizabeth E. Holland, Heidi R. Roth

**Affiliations:** Department of Physical Therapy and Human Movement Sciences, Feinberg School of Medicine, Northwestern University, 645 N. Michigan Avenue, Suite 1100, Chicago, IL 60611 USA

**Keywords:** Educational assessment, Physical therapist assistant education, Physical therapist education, Competency-based education, Assessment validity

## Abstract

**Background:**

High-quality educational assessments in health professions education are critical for ensuring that program graduates are competent to perform safe and effective patient care. While scoping reviews of available assessments and assessment tools have been conducted in some health professions, such knowledge synthesis is not available for physical therapy. The objective of this scoping review is to systematically describe the assessment of knowledge and skills in entry-level physical therapy education and identify gaps that need to be filled.

**Methods:**

The PubMed, CINAHL, ERIC, and Scopus electronic databases were searched from inception through April 2025. Studies were included if they evaluated an educational assessment of knowledge and/or skills in physical therapy students. Two reviewers independently screened the abstracts and full texts. One reviewer extracted information from each article, with verification from a second reviewer. Data charting included study characteristics, assessment characteristics, and validity evidence according to Messick’s validity framework. The level of evidence in each study was rated on a four-point scale. Fifteen physical therapy educators and five health professions education experts provided consultation to inform the interpretation of findings.

**Results:**

Of 4705 studies screened, 139 met the inclusion criteria. Ninety-two assessments were included, 74 of which were performance-based assessments. Clinical knowledge, procedural skills, and communication skills were the most prevalent construct types. Knowledge for Practice, Patient and Client Care & Services, and Communication were the most prevalent domains represented from the American Physical Therapy Association’s (APTA’s) Domains of Competence. Content evidence was the most prevalent type of validity evidence, reported for 64 (70.0%) of the assessments. The validity evidence ratings had median ratings of 0 or 1 for each category of validity evidence.

**Conclusion:**

This scoping review summarizes and synthesizes the evidence for assessments of knowledge and skills in entry-level physical therapy education. This review helps educators identify and select assessment tools in physical therapy education, reveals gaps in the educational assessment literature, and outlines recommendations for future research areas and approaches.

**Supplementary Information:**

The online version contains supplementary material available at 10.1186/s12909-026-08927-z.

## Introduction

In health professions education, accurate and meaningful educational assessment of learning is vital to ensure that trainees are able to provide optimal patient care. Assessment is especially important for implementation of learner- and outcome-centered educational approaches, such as competency-based education (CBE), which features “assessment practices [that] support and document the developmental acquisition of competencies” [1].^p. 1005^ The essential nature of high-quality assessment underscores the importance of understanding currently existing educational assessments and their validity evidence.

Health professions education scholars in some disciplines have addressed this need through scoping and systematic reviews of the literature. Previous investigations include reviews of patient note assessment [2], technology-enhanced simulation assessments [3, 4], undergraduate medical education assessments [5], surgical educational assessments [6, 7], and nursing assessments [8, 9]. These reviews identify assessments and synthesize the available validity evidence, providing a foundation for health professions educators and researchers to advance the state of assessment by mapping what is already known and outlining gaps that need to be filled.

Although some reviews of health professions educational assessments have included physical therapy [10, 11] or addressed skills that overlap with the scope of physical therapy practice (e.g., infection control procedures, cardiopulmonary resuscitation) [3], they do not represent most essential knowledge and skills for physical therapy practice. The absence of knowledge synthesis related to physical therapy-specific educational assessments hinders busy educators and researchers from selecting, interpreting, and using pre-existing evidence-based educational assessments. Knowledge of available assessments and their validity evidence is essential for entry-level physical therapy education programs to ensure that graduates are ready for independent, safe, and effective patient care. Filling this gap in the literature will enable physical therapy educators to effectively and efficiently use evidence-based educational assessments and assist researchers to identify where more research is needed.

**Fig. 1:**
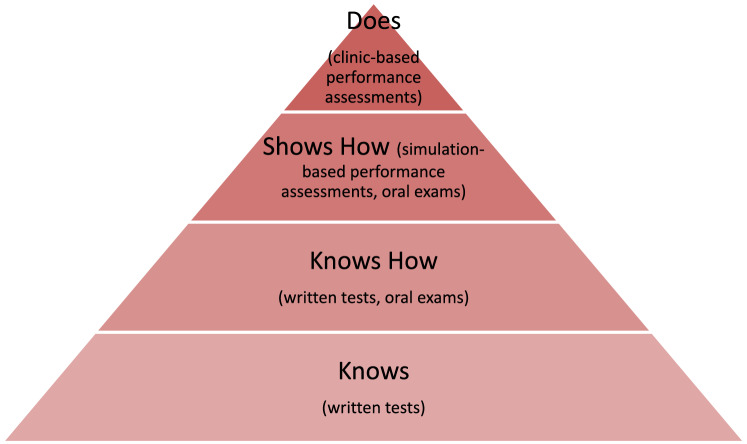
Miller’s pyramid with corresponding assessment formats [12, 13]

To understand the methods of assessment that are important for physical therapy education, Miller’s pyramid offers a useful framework (Fig. 1) [12, 13]. At the bottom of the pyramid, “Knows” refers to factual information that forms the foundation for higher levels of learning. This knowledge is best assessed with written exams. “Knows How” reflects more complex cognitive knowledge where the learner applies, analyzes, evaluates or constructs knowledge in a meaningful way. This level of knowledge is most appropriately tested with written or oral exams. “Shows How” applies knowledge and skill to a simulated situation and is typically assessed with performance-based assessments with standardized simulations or oral exams with standardized scenarios. At the top of Miller’s pyramid, the “Does” level reflects what the learner does in real-life scenarios such as patient care. This level is best assessed in clinic-based performance assessments. Miller’s pyramid provides a useful model for understanding the variety of knowledge and skill assessments in physical therapy education.

This scoping review addresses the question, “What are the characteristics of educational assessments of knowledge and skills that have been evaluated in entry-level physical therapy education?” The aims are to: (1) systematically describe the current state of the evidence for the assessment of knowledge and skills in physical therapy education and (2) identify gaps in assessment that need to be filled to ensure that physical therapist (PT) and physical therapy assistant (PTA) program graduates are competent to perform safe and effective patient care.

## Methods

Our aims for this review reflect a need for a map of the literature rather than seeking an answer to a narrow question, therefore we selected scoping review methodology [14]. This scoping review follows the Arksey and O’Malley framework [15], including updates from Levac et al. [16], through six steps: (1) research question identification, (2) relevant study identification, (3) study selection, (4) data charting, (5) results compilation, summarization, and reporting, and (6) consultation. Reporting of this research follows the Preferred Reporting Items for Systematic Reviews and Meta-Analysis – Extension for Scoping Reviews (PRISMA-ScR) [17]. Covidence review software (Veritas Health Innovation, Melbourne, Australia) was used for the study selection and data charting steps.

### Research question identification

Based on our experience as physical therapy educators and an initial review of the literature, we identified the following research question: “What are the characteristics of educational assessments of knowledge and skill by faculty and preceptors that have been evaluated in entry-level physical therapy education?” We were especially interested in the characteristics that might inform what the assessment scores mean and what summative decisions can reasonably be made based on the assessment results. Therefore, we identified assessment characteristics that are important for informing assessment interpretation and use, including target construct, assessment format, and validity evidence. We also focused our research question on assessments of knowledge and skill by faculty and preceptors to limit the synthesis to assessments that focus on students’ observable behaviors and therefore target assessments that serve a summative purpose.

### Relevant study identification

In consultation with a research librarian, the PubMed, CINAHL, ERIC, and Scopus databases were searched for English-language peer-reviewed references from database inception to July 28, 2023. A second search of the same four databases was conducted with the same search terms from 2023 through April 21, 2025. Search terms focused on: assessment, physical therapy, education, and student. Supplement 1 outlines the full search strategy. Inclusion criteria were articles of any single study type that have a stated purpose to evaluate an educational assessment of knowledge and/or skills in PT or PTA students. Articles were excluded if: (1) the full text was not available, (2) the article was a review, (3) the assessment was not intended for use within an entry-level PT or PTA curriculum (e.g., admissions assessments, licensure examinations, assessment of physical therapy graduates, residents, or fellows), (4) the assessment measured attitudes or traits rather than knowledge and/or skills, (5) the assessment was not rated by a preceptor (e.g., student self-assessment), (6) the assessment purpose was to measure program outcomes rather than individual students’ learning outcomes, (7) the report did not provide or reference sufficient description to allow replication of the assessment. When articles lacked sufficient description of the assessment, the corresponding author was contacted in an attempt to obtain this information. The reference lists from reviews that were excluded were scanned for additional relevant studies.

### Study selection

To enhance interrater reliability, title and abstract screening began with a calibration exercise where all authors independently screened 50 titles and abstracts, discussed any discrepancies and calculated percent agreement. This process was repeated until 75% agreement was achieved. The remaining titles and abstracts were independently screened for inclusion by two authors. If both reviewers agreed that the article was reasonably likely to meet the inclusion criteria or that there was insufficient information in the abstract, the article advanced to full-text review. Discrepancies were resolved through discussion, and the third author resolved any remaining discrepancies.

Full-text screening began with a calibration exercise with independent review of 20 articles at a time by all authors until 75% agreement was achieved. The remainder of the articles underwent independent review by two authors with resolution of discrepancies through discussion and, if needed, the third author. The full-text articles’ reference lists were scanned, and any previously unidentified, appropriate references were added to the review. Interrater agreement for study inclusion during full-text review was 86.8%, and chance-adjusted interrater agreement using Cohen’s kappa was 0.66 (substantial agreement) [18].

### Data charting

One author (RST) created the extraction tool, which was then piloted and refined by the author team using six articles during four meetings. One author extracted data from each of the remaining articles, and one other author verified the data extraction for each article with resolution of discrepancies through discussion and, if needed, the third author. Based on the interpretative nature of the data extraction related to validity evidence and Domains of Competence (described below), the authors met regularly to discuss discrepancies, review the frameworks underlying the extraction categories, and ensure adequate resolution of discrepancies.

Data charting included extraction of the following study characteristics: the country in which the study was conducted; the study design using criteria from the Medical Education Research Study Quality Instrument (MERSQI) [19]; assessment name; format; the assessment’s intended interpretation and use [20, 21]; target population; target constructs; and alignment of the assessment content with the American Physical Therapy Association (APTA) Domains of Competence [22, 23]. The APTA Domains of Competence were selected because of their relevance to the authors’ setting. Review of other countries’ competency frameworks reflects alignment with these domains [24]. Data extraction related to the APTA Domains of Competence incorporated the most recent version of the assessment (e.g., Clinical Performance Instrument (CPI) version 3.0 rather than the first version of the CPI). Data extraction also included information about assessment validity from each study, including the validity framework used, and the validity evidence presented for each assessment [20, 21, 25, 26].

In the field of educational measurement, scholars have shifted away from classical validity theory toward contemporary validity frameworks [21, 25]. The current *Standards for Educational and Psychological Testing* (the “*Standards”)* [20] are based in Messick’s [26, 27] and Kane’s validity frameworks [28, 29]. In these frameworks, validity is a characteristic of the proposed interpretation of scores (i.e., what do the scores mean? ) for a specific assessment use (i.e., what decisions will be made based on assessment scores? ) rather than a characteristic of the assessment tool itself. Thus, validity evidence is gathered to support or refute the argument for the assessment’s proposed interpretation and use. This view of validity contrasts with classical validity theory (i.e., content, criterion, and construct validity), where validity is framed as a characteristic of the assessment itself.

Data extraction included identification of the validity framework underlying each study, including classical validity theory, Messick’s framework, and Kane’s framework [20, 21, 25, 26, 28, 30, 31]. If the validity framework was not named, the framework was inferred when possible based on terminology and framing used in each article’s discussion of validity. The validity evidence in each article was categorized according to the five sources of validity evidence from Messick’s framework: content, response process, internal structure, relations with other variables, and consequences [4, 20, 27]. See Table 1 for definitions of each source. For each type of validity evidence, information about the validity investigation methods was extracted [3, 6], categorized using criteria adapted from Cook et al. [4], and rated on a 4-point scale of the strength of the evidence using criteria adapted from Ghaderi et al. [6] and Beckman et al. [32] (Table 2).

**Table 1:** The five sources of validity evidence

Source	Description	Examples
Content Evidence	Steps taken to ensure that the assessment accurately represents the target construct that the assessment is intending to measure.	• Test blueprint • Expert review of test content • Pilot testing with iterative revision of content
Response Process Evidence	Evidence that evaluates how well the test processes and rater and examinee responses match the intended construct.	• Test security measures • Investigation of raters’ or examinees’ thoughts during the assessment
Internal Structure Evidence	Evaluation of the relationship between the individual assessment items and the target construct.	• Reliability measures • Item analysis • Factor analysis
Relations with Other Variables Evidence	Associations between assessment scores and other measures that have a theoretical relationship. The association may be positive (for two measures that theoretically should be related) or negative (for two measures that theoretically should be different from each other).	• Correlation of assessment scores with future outcomes, such as licensure exam or patient care outcomes • Correlation between assessment scores and training level
Consequences Evidence	Evidence for the impact of an assessment and decisions that result from an assessment. The impact may be positive or negative, intended or unintended.	• Evaluating the impact of an assessment’s ability to accurately identify students in need of additional instruction. • Evaluating the impact of bias in assessment on examinees

The five sources of validity evidence from Messick’s validity framework [4] and the *Standards for Educational and Psychological Testing* [20] are listed and described with examples of evidence that would typically fall within each category

**Table 2:** Validity evidence rating scale

**Content Evidence**	
0	No data or evidence regarding the instrument content.
1	Limited amount of data to justify the instrument content.
2	Listing assessment themes with some references and justifications, limited description of the process for creating the instrument.
3	Well-defined process for developing instrument content, including both an explicit theoretical/conceptual basis for instrument items and systematic item review by experts.
**Response Process Evidence**	
0	No data or evidence regarding the response process.
1	Minimal data or evidence presented. Discussing the impact of response rate on assessment scores or speculating on the thought processes of learners.
2	Some data regarding thought processes and analysis of responses. Some data about implication of systems that reduced response error.
3	Multiple sources of supportive data, including critical examination of thought processes, analysis of responses for evidence of halo error or rater leniency, or data demonstrating low response error.
**Internal Structure Evidence**	
0	No data or evidence regarding internal structure.
1	Minimal data with regard to internal structure, some reliability with a single measure.
2	Factor analysis incompletely confirming anticipated data structure or a few measures of reliability reported.
3	Factor analysis confirming anticipated data structure or multiple measures of reliability, item analysis data, item/test characteristic curves (ICCs/TCCs), interitem correlations, item-total correlations), generalizability analysis.
**Relations with Other Variables Evidence**	
0	No data or evidence regarding relations with other variables.
1	Correlation of assessment scores to outcomes with minimal theoretical importance, a single measure of validity (relationship between level of training and scores).
2	Correlation of assessment scores to outcomes with some theoretical importance.
3	Correlation (convergence) or no correlation (divergence) between assessment scores and theoretically predicted outcomes or measures of the same construct. Such evidence will usually be integral to the study design and anticipated a priori, generalizability evidence.
**Consequences Evidence**	
0	No data or evidence regarding consequences.
1	Limited data about the consequences of the assessment. Merely discussion about the consequences of assessment (e.g., data regarding usefulness of assessment based on post-assessment survey).
2	Description of consequences of assessment that could conceivably impact the validity of score interpretations (although these impacts are not explicitly identified by the authors).
3	Description of consequences of assessment that clearly impact on the validity of score interpretations, as supported by data and convincingly argued by the authors. Such evidence will usually be integral to the study design and anticipated a priori.

The validity evidence in each article was rated for each of the five sources of validity evidence from Messick’s validity framework [25] using criteria adapted from Ghaderi et al. [6] and Beckman et al. [32]

### Results compilation, summarization, and reporting

Quantitative analysis was used to summarize and synthesize the extracted data, using descriptive statistics (frequency, mean with standard deviation for ratio/interval data, median with interquartile range (IQR) for ordinal data). All authors reviewed the extracted data independently and in discussion to identify themes addressing the research question.

### Consultation

In this phase, invested parties provided feedback about the scoping review findings [33]. Two experts in physical therapy education and three experts in educational assessments in other health professions reviewed the findings and answered the following questions:

1. Are you aware of any eligible studies or assessments that were not included in this scoping review?
2. How will the results of this scoping review impact the field of physical therapy education? (for experts in physical therapy education)
3. How do the results of this scoping review compare with the landscape of educational assessments in your field? (for experts outside of physical therapy)
4. What do you think the next steps should be for physical therapy education researchers?

We also presented the preliminary findings of this scoping review at the 2025 APTA Annual Physical Therapy Education Leadership Conference (ELC) [34] and solicited feedback from attendees via an online poll to answer the following questions:

1. How will the results of this scoping review impact your educational practice?
2. What do you think the next steps should be for physical therapy education researchers?

### Reflexivity

The authors are all faculty members and educators in the Doctor of Physical Therapy (DPT) program at Northwestern University with extensive clinical experience as PTs and experience designing, implementing, and studying assessments in physical therapy education. Two authors (HRR and RST) have published peer-reviewed articles in clinical assessment and have experience conducting systematic and scoping reviews. One author (RST) is currently pursuing a PhD in Health Professions Education with a focus on assessment. These experiences inform our belief in the high importance of knowledge synthesis and assessment validity for effective and fair educational practice and, thus, our interpretation of the results of this review.

## Results

**Fig. 2:**
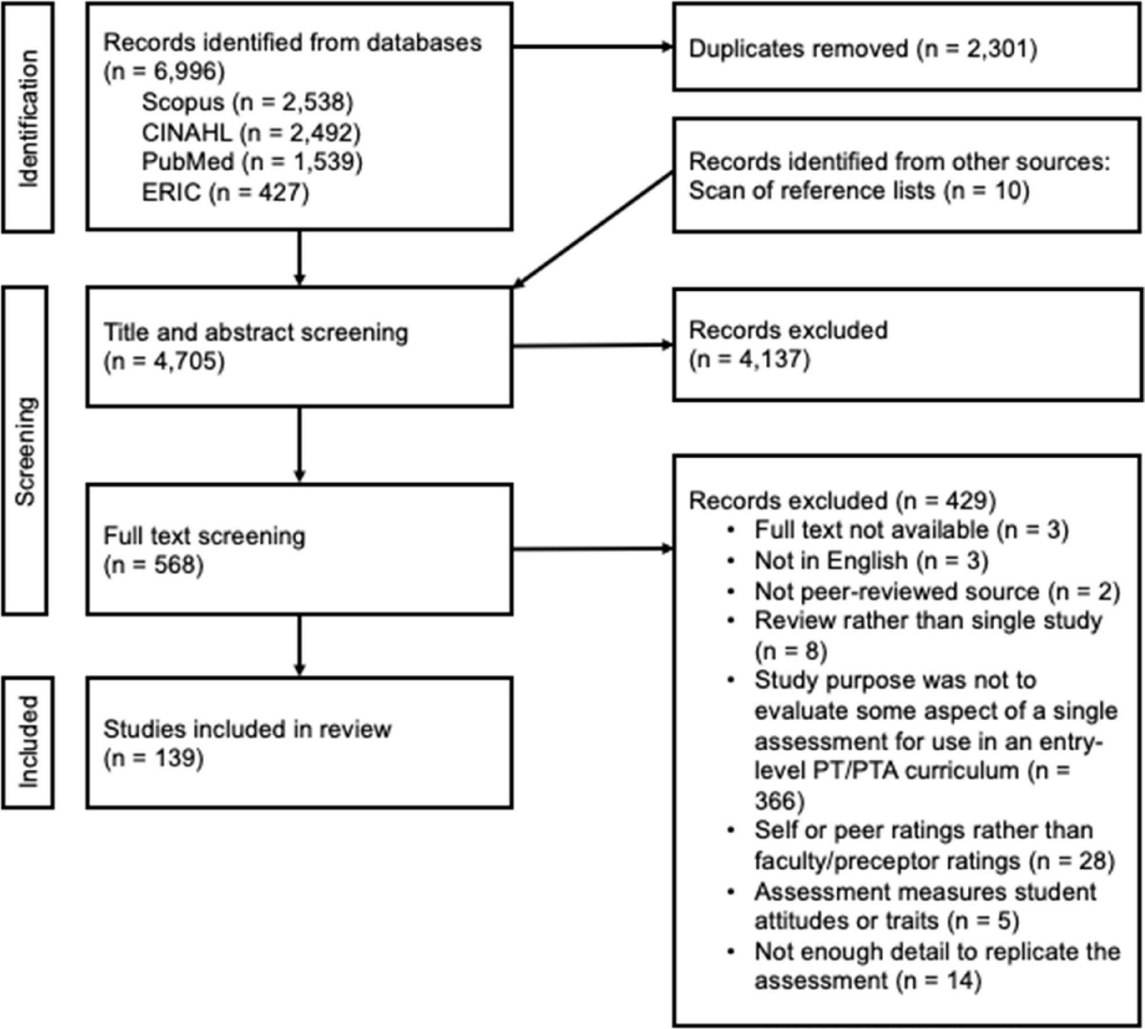
Preferred reporting items for systematic reviews and meta-analyses (PRISMA) flow diagram

The search identified 4705 studies after duplicates were removed, and 139 articles were ultimately included. See Fig. 2 for PRISMA flow diagram, Supplement 2 for a list of included articles, and Supplements 3–5 for summaries of data extraction results.

### Study characteristics

Most articles (*n* = 102, 73.4%) were published in 2010 or later. One hundred sixteen of the studies (83.5%) were single-group cross-sectional or single-group post-test only studies. The majority (*n* = 71, 51.1%) of studies were conducted in the United States, followed by Australia (*n* = 19, 13.7%) and Canada (*n* = 13, 9.4%). The remaining studies (*n* = 36, 25.9%) were conducted in 19 countries, with one to four studies in each country. The target population in each study was student PTs for most articles (*n* = 126, 90.6%), followed by interprofessional education (including student PTs or PTAs) (*n* = 7, 5.0%), multiple professions (including student PTs or PTAs, but not including interprofessional education) (*n* = 5, 3.6%), and a combination of student PTs and PTAs (*n* = 1, 0.7%).

### Assessment characteristics

Ninety-two distinct assessments were included in the articles, with multiple versions of an assessment (e.g., updated versions, translations) counted as a single assessment. Performance-based assessments were the most common format with 40 (43.5%) simulation-based assessments, 28 (30.4%) workplace-based assessments, two (2.2%) assessments intended for both the simulation and workplace environment, and four (4.3%) assessments that included performance-based assessments plus an additional format. The most common construct types were clinical knowledge, communication skill, and procedural skill. The APTA Domains of Competence that were most often addressed were: Knowledge for Practice, Patient/Client Care & Services, and Communication. Table 3 lists a summary of the characteristics of all the identified assessments. See also Table 4 for characteristics of the assessments that were reported in at least two of the included studies. Nineteen (13.7%) studies fully described the intended interpretation and use of the assessment.

**Table 3:** Characteristics of the assessments in the included studies

Assessment Format (by assessment)	Number of Assessments	
	*n*	%
Performance-based: Simulation	40	43.5%
Performance-based: Workplace	28	30.4%
Written	13	14.1%
Oral	3	3.3%
Performance-based: Simulation or Workplace	2	2.2%
Other	2	2.2%
≥ 1 Format	4	4.3%

**Assessment Construct Type** **(by assessment)**	**Number of Assessments**	
	**n**	**%**
Knowledge: Clinical	62	67.4%
Skill: Communication	62	67.4%
Skill: Procedural	56	60.9%
Skill: Teamwork	20	21.7%
Knowledge: Basic Science	6	6.5%
Skill: Other	4	4.3%
Knowledge: Other	1	1.1%
Open-ended	1	1.1%

**Domains of Competence (by assessment)**	**Number of Assessments**	
	**n**	**%**
Knowledge for Practice	79	85.9%
Patient and Client Care & Services	70	76.1%
Communication	65	70.7%
Professionalism	40	43.5%
Practice Management	35	38.0%
Reflective Practice & Improvement	26	28.3%
Education & Learning	20	21.7%
Systems Based Practice in Healthcare	6	6.5%

Assessment format reflects the main format(s) of the assessment design. Assessment construct type reflects the type of knowledge and/or skills that are targeted for assessment. Many of the assessments targeted more than one type of construct (e.g., clinical knowledge and procedural skill) and/or more than one Domain of Competence

**Table 4:** Commonly reported assessments in physiotherapy education (i.e., assessments with more than one validation study)

Assessment Name	Assessment Format	Primary Construct Tested	Total # of Studies (*n*)	Validity Evidence														
				Content			Response Process			Internal Structure			Relations with Other Variables			Consequences		
				**# Studies (n)**	Min Rating	Max Rating	**# Studies (n)**	Min Rating	Max Rating	**# Studies (n)**	Min Rating	Max Rating	**# Studies (n)**	Min Rating	Max Rating	**# Studies (n)**	Min Rating	Max Rating
Assessment of Physiotherapy Practice (APP)	Performance – Workplace-based	Clinical Competence	12	**5**	2	2	**3**	1	2	**8**	1	3	**3**	1	1	**4**	1	2
Canadian Physiotherapy Assessment of Clinical Performance (ACP)	Performance – Workplace-based	Clinical Performance	5	**2**	2	3	**4**	1	3	**1**	1	1	**1**	3	3	**1**	1	1
Clinical Internship Evaluation Form (CIET)	Performance – Workplace-based	Clinical Performance	3	**3**	1	3	**2**	2	2	**2**	1	2	**2**	1	2	**2**	1	1
Clinical Performance Assessment Form	Performance – Workplace-based	Clinical Performance	2	**1**	2	2	**0**	N/A			2	2	**0**	N/A		**0**	N/A	
Clinical Performance Instrument (CPI)	Performance – Workplace-based	Clinical Performance	15	**2**	2	3	**6**	1	2	**7**	1	3	**8**	1	3	**0**	N/A	
Clinical Reasoning Assessment Tool (CRAT)	Oral Performance – OSCE/Simulation and Workplace-based	Clinical Reasoning	4	**1**	3	3	**2**	1	2	**1**	2	2	**1**	1	1	**1**	1	1
Common Assessment Form (CAF)	Performance – Workplace-based	Clinical Performance	2	**2**	2	2	**1**	2	2	**1**	1	1	**1**	2	2	**0**	N/A	
ECHOWS Tool	Performance – OSCE/Simulation and Workplace-based	Patient Interview Skills	2	**0**	N/A		**1**	1	1	**2**	1	2	**1**	1	1	**0**	N/A	
Evaluation of Clinical Competence (ECC)	Performance – Workplace-based	Clinical Competence	2	**2**	2	3	**0**	N/A		**1**	1	1	**1**	2	2	**0**	N/A	
Integrated Standardized Patient Examination	Performance – OSCE/Simulation	Clinical Competence	3	**2**	1	1	**0**	N/A		**2**	2	3	**1**	1	1	**1**	2	2
Modified Fresno Test	Written – Essay	Evidence Based Practice	4	**3**	3	3	**1**	2	2	**4**	1	3	**1**	1	1	**1**	1	1
Musculoskeletal OSCE	Performance – OSCE/Simulation	Musculoskeletal Competence	2	**1**	1	1	**1**	2	2	**2**	2	2	**0**	N/A		**1**	2	2
Physical Therapist Manual for the Assessment of Clinical Skills (PT MACS)	Performance – Workplace-based	Clinical Performance	2	**1**	3	3	**0**	N/A		**1**	1	1	**1**	2	2	**0**	N/A	
Unnamed OSCE from Figueroa-Arce 2022 and Figuero-Gonzalez 2023	Performance – OSCE/Simulation	Clinical Reasoning	2	**2**	1	2	**0**	N/A		**0**	N/A		**1**	1	1	**2**	1	1
Unnamed assessment from Garcia-Ros 2021 and 2024	Performance – OSCE/Simulation	Neurologic Physiotherapy Skills (NDT/PNF)	2	**2**	2	2	**0**	N/A		**1**	3	3	**0**	N/A		**2**	1	1

The validity evidence in each article for each of the five sources of validity evidence were rated on a scale from 0 to 3 using criteria adapted from Ghaderi et al. [6] and Beckman et al. [32]. The number of studies that include each type of validity evidence are highlighted in bold. See Table 2 for a detailed description of the rating scale

### Validity evidence - overview

Seven (5.0%) studies used a contemporary framework for educational assessment validity, (i.e., Messick’s framework [27], Kane’s framework [29], or a combination of the two frameworks as described in the *Standards for Educational and Psychological Testing* [20]). The remaining studies used the classical validity model (*n* = 89, 64.0%) or did not describe assessment validity in a way that allowed a determination of the framework used (*n* = 43, 30.9%). Table 4 outlines the range of validity evidence levels present in the included studies for each of the more commonly studied assessments. In the following sections, we will summarize the types and quality of validity evidence identified for each of the five sources of validity evidence within Messick’s framework to highlight areas of strength and opportunities for improvement.

### Validity evidence - content

See Table 1 for a definition of content evidence and Table 2 for definitions of the content evidence ratings. Content evidence was included in 78 (56.1%) studies, covering 64 (70.0%) of the 92 identified assessments. The highest level of content evidence reported for each assessment had a median rating of 1 (IQR = 0, 2). The most common type of data was from expert panels including Delphi reviews, surveys, and interview methods, which was present in 58 (41.7%) studies for 48 (52.2%) of the assessments. Other types of data identified were: adaptation from previous instruments, pilot testing and revision, use of guidelines to determine content, rigorous scoring development, and use of a test blueprint to determine test content. See Supplement 3a for frequencies of each data type.

### Validity evidence - response process

See Table 1 for a definition of response process evidence and Table 2 for definitions of the response process evidence ratings. Response process evidence was included in 43 (30.9%) studies, covering 32 (34.8%) of the identified assessments. The highest level of response process evidence reported for each assessment had a median rating of 0 (IQR = 0, 2). The most common type of data was evaluation of rater and examinee perceptions of the assessment processes, which was present in 21 (15.1%) studies and covered 18 (20.0%) of the identified assessments. See Supplement 3b for frequencies of each data type.

### Validity evidence - internal structure

See Table 1 for a definition of internal structure evidence and Table 2 for definitions of the internal structure evidence ratings. Internal structure evidence was included in 70 (50.4%) studies, covering 49 (53.2%) of the identified assessments. The highest level of internal structure evidence reported for each assessment had a median rating of 1 (IQR = 0, 2). Reliability was the most common type of data, present in 59 (42.4%) studies and covering 44 (47.8%) of the identified assessments. Specific types of reliability represented included (in order of prevalence): interrater reliability, internal consistency, intrarater reliability, test-retest reliability, and analysis of variance across facets. See Supplement 3c for frequencies of each data type.

### Validity evidence - relations to other variables

See Table 1 for a definition of relations with other variables evidence and Table 2 for definitions of the relations with other variables evidence ratings. Relations with other variables data was included in 47 (33.8%) studies, covering 37 (40.2%) of the identified assessments. The highest level of relations to other variables evidence reported for each assessment had a median rating of 0 (IQR = 0, 1). With relations to other variables evidence, associations (usually in the form of statistical correlations) are made with other variables. In these studies, learner characteristics (e.g., level of training) were the most prevalent type of comparison variable, followed by concurrent measures and delayed measures. See Supplement 3d for frequencies of each data type.

### Validity evidence - consequences

See Table 1 for a definition of consequences evidence and Table 2 for definitions of the consequences evidence ratings. Consequences data was included in 36 (25.9%) studies, covering 31 (33.7%) of the identified assessments. The highest level of content evidence reported for each assessment had a median rating of 0 (IQR = 0, 1). Specific types of data that were represented include: standard setting methods, evaluation of pass rates, and evaluation of anticipated and unanticipated impact of the assessments. See Supplement 3e for frequencies of each data type.

### Consultation

The expert physical therapy educator consultants both currently teach in entry-level DPT programs. One consultant oversees the curriculum in a DPT program and has held multiple leadership positions in their home institution and the APTA’s Academy of Physical Therapy Education. The other consultant has a PhD in Educational Psychology with an emphasis in statistics and measurement. In their responses to the consultation questions, they both highlighted the benefit of educators having access to a list of available assessments and summaries of each assessment to assist in assessment identification and selection. They also noted that the scoping review findings identify and address gaps in the physical therapy education literature, including investigating needed evidence for educational assessments and advancing the discourse around assessment validity to include contemporary frameworks of validity. They highlighted the need for additional professional development for physical therapy educators and researchers to advance assessment practices and research.

The experts in medical education assessment are all established, recognized scholars of assessment in medical education with extensive publications and national and international presentations. The experts agreed that the findings in the physical therapy literature were mostly aligned with the medical education literature regarding the assessment formats and types of validity evidence represented for single assessments. One consultant noted that contemporary validity frameworks, including the Messick and Kane frameworks, are more predominant in the medical education literature than in physical therapy education, based on the results of this scoping review. They also noted a higher prevalence of written assessments, portfolio assessments, and assessments based on Entrustable Professional Activities (EPAs) in the medical education literature. They recommended that physical therapy researchers also address: assessment systems and programs, the ways in which individual assessment fit into larger programs of assessment, assessment of longitudinal performance, use of qualitative assessment data, and incorporating artificial intelligence into the process of interpreting assessment data.

Fifteen ELC presentation attendees responded to the online poll. The themes noted in the responses were: (1) the scoping review results will help educators select the most appropriate educational assessments, and (2) it would be helpful for physical therapy educators and researchers to have additional training in and resources for use of contemporary validity frameworks for educational assessment.

## Discussion

This scoping review maps the validity evidence for educational assessments in entry-level physical therapy education to summarize the available evidence for use in educational practice and to outline the gaps in evidence to guide future research. We found that performance-based assessments intended for use in clinical education and in simulation were by far the most studied type of assessment, representing 80.4% of the assessments included in this scoping review and 93.3% of the 15 assessments with more than one validation study. These assessments correspond to the top two layers of Miller’s pyramid: “Does” and “Shows How” (Fig. 1). Clinical knowledge, procedural skills, and communication skills were the most highly represented construct types, corresponding to the “Does,” “Shows How,” and “Knows How” ability levels from Miller’s pyramid. Knowledge for Practice, Patient and Client Care & Services, and Communication were the APTA Domains of Competence that were most often addressed by the included assessments. Based on their direct connection with patient care, these constructs and APTA Domains of Competence align with the high prevalence of clinic-based and simulation-based assessments. The “Knows” and to a lesser extent the “Knows How” levels of Miller’s pyramid were underrepresented with few published assessments reflecting written and oral exams covering knowledge-based content.

### Comparison to other health professions

The high prevalence of performance-based assessments was consistent with scoping reviews in physician [3, 5, 6] and nursing [8] education. Of these scoping reviews, Song et al.’s review of assessments in undergraduate medical education was most analogous to the entry-level physical therapy education setting. A notable difference in the findings between Song et al.’s findings and this review is that there was a higher prevalence of written assessments in the undergraduate medical education literature. This difference is likely related to the higher prevalence of standardized written assessments implemented across programs during undergraduate medical training (i.e., United States Medical Licensing Examination (USMLE) Step exams, National Board of Medical Examiners (NBME) subject exams).

The representation of the APTA Domains of Competence in physical therapy educational assessments aligns with that found in undergraduate medical education. In their review of undergraduate medical education assessments, Song et al. found that the competencies of Patient Care and Medical Knowledge were the most frequently assessed domains [5]. These domains are analogous to the APTA’s Patient and Client Care & Services and Knowledge for Practice domains, which were the most frequent domains in this review. Song et al. found that the Systems-based Practices and Practice-based Learning were the least assessed domains. Similarly, these domains are analogous to the APTA’s Systems-based Practice in Healthcare and Reflective Practice & Improvement, which were the least and third least commonly addressed domains in this review respectively.

### Validity evidence - gaps

The findings of this scoping review highlight the need for educational assessments with more robust validity evidence. While content and internal structure validity evidence were frequently represented, less than half of the assessments had any validation study that investigated response process, relations with other variables, or consequences evidence. Furthermore, the ratings of the highest level of evidence in each category across assessments had a median of 0 or 1, highlighting substantial opportunity to increase the strength of assessment validity evidence in our field. For instance, expanding the variety of investigation methods used to examine internal structure is one opportunity. Also, more investigation of the relationship between assessment scores and future outcomes as well as the anticipated and unanticipated impact of assessments would lead to important increases in relations with other variables evidence and consequences evidence, respectively. These gaps in the type and quality of validity evidence call into question whether interpretations of student ability and decisions about student progression based on the assessment scores are justified.

The included studies overwhelmingly used the classical validity framework, which is no longer considered best practice for educational assessments [20]. Classical validity theory is ubiquitous in clinical research [35–40] and therefore familiar and easy to use for educators coming from a clinical background. However, in the field of education classical validity has been replaced by contemporary validity frameworks that shift the focus from validity as a property of the assessment to a characteristic of the intended interpretation and use of an assessment [20, 21, 25, 27]. For example, if assessment scores will be used to determine whether a trainee is ready for independent patient care, the validity evidence needed to justify the assessment interpretation and use will be different in type and amount compared to if the assessment will be used for residency selection or as a formative quiz. Therefore, according to contemporary validity theory, the argument for assessment validity must be framed in relationship to its intended interpretation and use. Clinical research partially accounts for this limitation in classical validity theory by acknowledging that the presence of validity evidence in one patient population does not necessarily mean that an outcome measure is “validated” in a different patient population [35–37]. Contemporary validity theory in education substantially extends this idea. A detailed discussion of contemporary validity theory is beyond the scope of this article, and we encourage readers to refer to key references about validity and educational assessment [20, 25, 27, 30].

The predominant use of the classical validity framework was associated with missing information about the assessments being studied. Critical steps in assessment validation according to contemporary validity theory are: (1) specifying the intended interpretation and use of the assessment, and (2) gathering empirical data or theoretical arguments that support or refute the assessment’s interpretation and use [26]. However, only 13.7% of the studies fully reported the intended ways in which the assessment would be interpreted and what decisions would result from the assessment – two elements that fundamentally shape what type and amount of validity evidence is necessary [20, 21, 25]. Furthermore, only 5% of the studies framed the evidence for the assessment’s interpretation and use using a contemporary validity framework. These findings represent a major gap in the physical therapy education literature relative to best practices in the broader field of education.

Improving the state of the evidence for physical therapy educational assessments requires that we, as a profession, address the underlying causes of these shortcomings. While we do not yet have research evidence to guide this process, we offer our observations to start the conversation. Educational assessment has historically received less emphasis within physical therapy education than other health professions. For instance, we notice that assessment-related content is less common in physical therapy-specific education journals and conferences than in medical education spaces. Physical therapy education programs are also less commonly associated with health professions education scholarship units [41] compared to medical education programs. As a result, the access to experts with formal research training in educational assessments is lower in physical therapy, resulting in less methodological expertise and opportunities for research collaborations.

### Implications for educational practice

Our findings reveal multiple gaps in the physical therapy education literature: underrepresented areas of knowledge and skills assessment, validation approaches that are inconsistent with current education standards, and low levels of validity evidence. These limitations in the literature reflect a need for the physical therapy education community to update and deepen our understanding of critical concepts and best practices in educational assessment. Implementation of evidence-based assessments is essential to build the strong programs of assessment that are a core component of CBE [1].

This scoping review can help physical therapy educators identify and select educational assessment tools, a benefit that is in alignment with the findings from the consultation phase. We recommend that educators identify the purpose of the assessment first: What construct do they aim to measure? What interpretations do they intend to make about the students’ knowledge and skills based on the assessment scores? And what decisions will they make as a result of the assessment? Educators should then select an assessment tool based on the target construct, assessment type, and validity evidence that are the best match for the assessment purpose. For instance, if the purpose of a simulation-based assessment is to determine whether students are ready to proceed to direct patient care in clinical education, relations with other variables evidence demonstrating an association between assessment performance and future performance with patients would be high-priority validity evidence [30, 42]. Educators should also consider gathering their own empirical or theoretical evidence for their local context [30]. Supplement 4 (a list of the included studies with assessment characteristics and validity evidence) and Supplement 5 (a list of the included assessments with related APTA Domains of Competence) can facilitate this process.

### Implications for research

Advancing the state of educational assessment in physical therapy will require considerable change in education research priorities and practices. Based on the findings of this review, including the results of the consultation phase, we recommend that physical therapy education researchers conduct validation studies for a wide variety of assessments. We have a responsibility to patients and society to ensure that our graduates are competent, and assessment is the means by which we accomplish this goal [43]. Therefore, validity evidence for both formative and summative assessment is a high priority [42]. For physical therapy programs utilizing a CBE approach, we must ensure that the assessment validity evidence sufficiently covers all locally relevant Domains of Competence and EPAs [22–24, 44].

In addition, best practices in assessment require many datapoints from a variety of assessments and assessment methods for reliable and valid decision-making about learner progression [42, 45, 46]. Systems of assessment, such as programmatic assessment (a core component of CBE [1]), rely on high-quality individual assessments [42]. The findings of this scoping review reveal the need for a heightened level of investigation and discourse on this topic. Other assessment research topics that are ripe for investigation include: the interpretation and use of qualitative assessment data and incorporating artificial intelligence into the process of interpreting assessment data.

We also recommend that all health professions education researchers transition to the validity framework described in the current *Standards for Educational and Psychological Testing* [20]. This transition will bring health professions education, including physical therapy, in closer philosophical and practical alignment with the broader education community, including the National Council on Measurement in Education and the American Educational Research Association. We recognize that the clinical research community continues to use the classical validity framework. Therefore, we challenge health professions education researchers to achieve fluency in both frameworks. We believe that advancing our conceptualization of validity is worth the effort.

### Limitations

This scoping review has limitations. Despite broad search strategies, we may have missed relevant studies. We limited our search to assessments of knowledge and skills implemented by faculty raters during entry-level training, which excluded affective domain assessments and self-assessments. We also limited our search to assessments where sufficient description of content and scoring was available to replicate the assessment. While this restriction allowed us to map the peer-reviewed literature that would be accessible and implementable for most educators, it excluded some assessment evidence in the literature as well as locally developed, unpublished assessment evidence. In addition, the data charting process combined with sometimes limited descriptions in the included articles required reliance on the raters’ judgements, e.g., about validity evidence and APTA Domains of Competence. We minimized bias related to these judgements through the calibration and data checking methods described in the Methods section.

Our extraction of information about the APTA Domains of Competence reflects which domains were at least partially addressed by each assessment. Questions remain about how fully the assessments cover the entire APTA Domains of Competence. This scoping review did not include data related to the recently published APTA Core Entrance-to-Practice Competencies and EPAs [23]. Organizing systems of assessment around the units of work represented in the EPAs should be the target of future work.

## Conclusion

This scoping review maps the current landscape of assessment validity evidence in the field of physical therapy education. While numerous assessments and validation studies exist, substantial gaps in the amount and levels of validity evidence remain. In addition, we found that the educational assessment discourse in physical therapy is mainly centered in classical validity frameworks rather than updated frameworks from the field of education. In this paper, we offer a synthesis of the available educational assessments in physical therapy that can be useful for educators and researchers. We also provide recommendations for future development in physical therapy education, including conducting validation studies for a wide variety of assessments and transitioning to contemporary frameworks for educational assessment validity.

## Supplementary Information

Supplementary Material 1. Title and Description: Scoping Review Supplement 1 Search Strategy. Search terms and strategy used for relevant study identification.

Supplementary Material 2. Title and Description: Scoping Review Supplement 2 List of included articles. List of included studies after study selection step.

Supplementary Material 3. Title and Description: Scoping Review Supplement 3 Frequency of Data Elements. Validity evidence data elements and prevalence for each of the five sources of validity evidence. Five tables list the types of validity evidence data elements identified and the prevalence of each data element for each type of validity evidence: content, response process, internal structure, relations with other variables, consequences.

Supplementary Material 4. Title and Description: Scoping Review Supplement 4 List of Articles with Assessment and Validity Evidence Characteristics. Included studies with assessment and validity evidence characteristics. Evidence ratings range 0-3 and are listed for each source of validity evidence. See Table 2 for validity evidence rating anchors. Reference list is included at the end of this document.

Supplementary Material 5. Title and Description: Scoping Review Supplement 5 List of Assessments with Assessment Characteristics. Included assessments with characteristics, including represented APTA Domains of Competence. For assessments with multiple versions, the Domains of Competence represented in the most recent English language version of the assessment are listed. Reference list is included at the end of this document.

## Data Availability

The data that support the findings of this study are available in the supplementary materials and from the corresponding author upon reasonable request.

## Abbreviations

APTA: American Physical Therapy Association
CBE: Competency-based education
CPI: Clinical Performance Instrument
DPT: Doctor of Physical Therapy
ELC: APTA Annual Physical Therapy Education Leadership Conference
EPA: Entrustable Professional Activity
IQR: Interquartile Range
MERSQI: Medical Education Research Study Quality Instrument
NBME: National Board of Medical Examiners
PRISMA-ScR: Preferred Reporting Items for Systematic Reviews and Meta-Analysis – Extension for Scoping Reviews
PT: Physical therapist
PTA: Physical therapist assistant
USMLE: United States Medical Licensing Examination
