# Supplementary material for: Educational assessments in entry-level physical therapy education: a scoping review
Source: BMC Med Educ. 2026 Mar 5;26:592. doi: 10.1186/s12909-026-08927-z (PMC13069796; doi:10.1186/s12909-026-08927-z)
Supplement: Supplementary file 1 — Supplementary Material 1. Title and Description: Scoping Review Supplement 1 Search Strategy. Search terms and strategy used for relevant study identification. [file 12909_2026_8927_MOESM1_ESM.docx]

Additional File 1: Search Strategy

As of 7/28/2023

Research question/topic: What are the characteristics of educational assessments of knowledge and skill that have been evaluated in entry-level physical therapy education?

Concept A: assessments

Concept B: physical therapy

Concept C: education

Concept D: Student

Search strategy

Concept A: Assessments

**MeSH**

- "Educational Measurement"[Mesh]

**Keywords**

- Evaluation
- Assessment
- Competency
- Measurement

**PubMed Search String**

"Educational Measurement"[Mesh] OR Evaluat*[title/abstract] OR Assessment*[title/abstract] OR Competenc*[title/abstract]

**General Search String**

Evaluat* OR Assessment* OR Competenc* OR Measurement*

Concept B: Physical therapy

**MeSH**

"Physical Therapy Modalities"[Mesh] OR "Physical Therapy Specialty"[Mesh]

**Keywords**

- Physical therap*
- Physiotherap*

**PubMed Search String**

"Physical Therapy Modalities"[Mesh] OR "Physical Therapy Specialty"[Mesh] OR “Physical therap*”[tiab] OR Physiotherap*[tiab]

**General Search String**

Physical therap* OR Physiotherap*

Concept C: Education

**MeSH**

- "Education, Professional"[Mesh]

**Keywords**

- Education
- Curriculum
- Practicum
- Residency
- Internship

**PubMed Search String**

"Education, Professional"[Mesh] OR Education*[tiab] OR Curriculu*[tiab] OR Practicum*[tiab] OR Residency[tiab] OR Internship*[tiab]

**General Search String**

Education* OR Curriculu* OR Practicum* OR Residency OR Internship*

**PubMed Search – Copy and paste into PubMed**

(“Educational Measurement”[Mesh] OR Evaluat*[title/abstract] OR Assessment*[title/abstract] OR Competenc*[title/abstract])

**AND**

(“Physical Therapy Specialty”[Mesh] OR “Physical therap*”[tiab] OR Physiotherap*[tiab])

**AND**

(“Education, Professional”[Mesh] OR Education*[tiab] OR Curriculu*[tiab] OR Practicum*[tiab] OR Internship*[tiab])

**AND**

("Students"[Mesh] OR student*[tiab] OR intern[tiab] OR interns[tiab])

**General Search– can copy and paste into any database.**

(Evaluat* OR Assessment* OR Competenc* OR Measurement*)

**AND**

(Physical therap* OR Physiotherap*)

**AND**

(Education* OR Curriculu* OR Practicum* OR Internship*)

**AND**

(Student* OR intern OR interns)

---

**Scopus**

TITLE-ABS-KEY ( ( evaluat* OR assessment* OR competenc* OR measurement* ) AND ( physical AND therap* OR physiotherap* ) AND ( education* OR curriculu* OR practicum* OR internship* ) AND ( student* OR intern OR interns ) )

Filters:

document type: article and review;

language: english
