# Supplementary material for: Educational assessments in entry-level physical therapy education: a scoping review
Source: BMC Med Educ. 2026 Mar 5;26:592. doi: 10.1186/s12909-026-08927-z (PMC13069796; doi:10.1186/s12909-026-08927-z)
Supplement: Supplementary file 2 — Supplementary Material 2. Title and Description: Scoping Review Supplement 2 List of included articles. List of included studies after study selection step. [file 12909_2026_8927_MOESM2_ESM.docx]

Additional File 2: List of included studies.

| **#** | **Citation** |
| --- | --- |
| 1 | Adams CL; Glavin K; Hutchins K; Lee T; Zimmerman C. An evaluation of the internal reliability, construct validity, and predictive validity of the Physical Therapist Clinical Performance Instrument (PT CPI). *Journal of Physical Therapy Education*. 2008;22(2):42-50. |
| 2 | Alpine LM, O'Connor A, McGuinness M, Barrett EM. Performance-based assessment during clinical placement: Cross-sectional investigation of a training workshop for practice educators. *Nurs Health Sci.* 2021;23(1):113-122. doi: 10.1111/nhs.12768. Epub 2020 Sep 9. PMID: 32803810. |
| 3 | Balaraman T, Śliwmski Z. The effectiveness of a case based online video assignment in improving practical performance of physiotherapy students - a quasi experimental study. *Fizjoterapia Polska*. 2021;21(2):30-34. |
| 4 | Bayliss J, Thomas RM, Eiffert-Mangine M. Pilot study: What measures predict first time pass rate on the National Physical Therapy Examination? *Internet Journal of Allied Health Sciences and Practice*. 2017;15(4):Article 1. |
| 5 | Becker M, Shields RK, Sass KJ. Psychometric analysis of an integrated clinical education tool for physical therapists. *J Phys Ther Educ*. 2024;38(4):277-284.  DOI: 10.1097/JTE.0000000000000341 |
| 6 | Birkmeier M, Wheeler E, McGregor Garske H, et al. Feasibility of use of the Clinical Internship Evaluation Tool in full-time clinical education experiences: A multi-institutional study. *Journal of Physical Therapy Education.* 2022;36(3):264-271. |
| 7 | Boissonnault JS, Evans K, Tuttle N, et al. Reliability of the ECHOWS tool for assessment of patient interviewing skills. *Phys Ther.* 2016;96(4):443-455. |
| 8 | Boruff JT, Thomas A. Integrating evidence-based practice and information literacy skills in teaching physical and occupational therapy students. *Health Information and Libraries Journal*. 2011;28:264-272. doi: 10.1111/j.1471-1842.2011.00953.x |
| 9 | Campbell DF, Alameri M, Macahilig-Rice F, Witkin SE, Hellman NG. Validation of the Revised American Physical Therapy Association Physical Therapist Clinical Performance Instrument 3.0. *Phys Ther*. 2025;105:pzaf015. doi: 10.1093/ptj/pzaf015 |
| 10 | Çelik Hľ, Ozturk D, Sari M, Bulut N, Bek N. Psychometric and edumetric properties of the Turkish version of the assessment of physiotherapy practice. *BMC Med Educ.* 2024;24(1):1162. doi: 10.1186/s12909-024-06180-w |
| 11 | Chapman JA, Westmorland MG. The structured oral self-directed learning evaluation: One method of evaluating the clinical reasoning skills of occupational therapy and physiotherapy students. *Medical Teacher.* 1993;15(2/3):223. |
| 12 | Chen LY, Lue YJ, Wu PH, Kuo YL. Cross-cultural adaptation and validation of the Chinese version of the modified Fresno test for physical therapists. *BMC Medical Education.* 2025;25(1):1. doi: 10.1186/s12909-024-06615-4 |
| 13 | Chong DYK, Tam B, Yau SY, Wong AYL. Learning to prescribe and instruct exercise in physiotherapy education through authentic continuous assessment and rubrics. *BMC Medical Education.* 2020;20:258. doi: 10.1186/s12909-020-02163-9 |
| 14 | Clark CR, Bialocerkowski A. Enhancing entry-level physiotherapy student learning in interpreting radiology - An action research project. *Internet Journal of Allied Health Sciences and Practice.* 2016;14(4):Article 5. |
| 15 | Coote S, Alpine L, Cassidy AC, et al. The development and evaluation of a Common Assessment Form for physiotherapy practice education in Ireland. *Physiotherapy Ireland.* 2007;28(2):6-10. |
| 16 | Costello E, Plack M Maring J. Validating a standardized patient assessment tool using published professional standards. *Journal of Physical Therapy Education.* 2011;25(3):30-46. |
| 17 | Cross V, Hicks, C, Barwell F. Exploring the gap between evidence and judgement: Using video vignettes for practice-based assessment of physiotherapy undergraduates. *Assessment & Evaluation in Higher Education.* 2001;26(3):189-212. |
| 18 | Dalton M, Davidson M, Keating J. The Assessment of Physiotherapy Practice (APP) is a valid measure of professional competence of physiotherapy students: A cross-sectional study with Rasch analysis. *Journal of Physiotherapy*. 2011;57:239-246. |
| 19 | Dalton M, Davidson M, Keating JL. The Assessment of Physiotherapy Practice (APP) is a reliable measure of professional competence of physiotherapy students: A reliability study. *Journal of Physiotherapy*. 2012;58:49-56. |
| 20 | Daly FJ. Use of electronic anatomy practical examinations for remediating "at risk" students. *Anatomical Sciences Education.* 2010;3(1):46-49. |
| 21 | Dickinson R, DiMarino J, Pfitzenmaier J. A common evaluation instrument. *Physical Therapy*. 1973;53(10):1075-1080. |
| 22 | Dorsey LL, Kelly PV, Luetkemeyer PB, Lojovich JM. Use of an academic professional behavior assessment and intervention to promote professional socialization. *Journal of Allied Health.* 2018;47(3):210-216c. |
| 23 | East L, Stevens A, Hageman H, Perlmutter M, Garuba F, Burlis T, Chang D, McConachie A, Hanson, J. Validity evidence for a feedback tool for an interprofessional standardized patient experience: A qualitative pilot study with evaluation of content, response processes, consequences of testing, and feedback quality. *Journal of Interprofessional Education and Practice*. 2025;40:100749. doi: 0.1016/j.xjep.2025.100749 |
| 24 | Edgeworth Ditwiler R, Swisher LL, Reddien Wagner B, Anderson SA. A model for professionalism evaluation: Using the RISE Assessment Tool across DPT didactic and integrated clinical education. *The Internet Journal of Allied Health Sciences & Practice*. 2023;22(1):Article 8. |
| 25 | Emmert MC, Cai L. A pilot study to test the effectiveness of an innovative interprofessional education assessment strategy. *Journal of Interprofessional Care.* 2015;29(5):451-456. |
| 26 | Fabrizio PA. Oral anatomy laboratory examinations in a physical therapy program. *Anat Sci Educ.* 2013;6:271-276. |
| 27 | Figueroa-Arce N, Figueroa-González P, Gómez-Miranda L, Gutiérrez-Arias R, Contreras-Pizarro V. [Implementation of an Objective structured clinical examination (OSCE) as a tool to evaluate the development of clinical reasoning in physical therapy students]. *Rev Fac Med*. 2022;70(2):e90746. English. doi: https://doi.org/10.15446/revfacmed.v70n2.90746 |
| 28 | Figueroa-González P, Figueroa-Arce N, Gómez-Miranda L, Gutiérrez-Arias R, Contreras-Pizarro V. Satisfaction level and correlation between performance and self-evaluation of physical therapy students in an objective structured clinical examination (OSCE) designed to assess clinical reasoning. *Revista Facultad de Medicina.* 2023;71(4):e107397. doi: 10.15446/revfacmed.v71n4.107397 |
| 29 | Fisher KA, Shobeiri SA, Nihira MA. The use of standardized patient models for teaching the pelvic floor muscle examination. *J Pelvic Med Surg.* 2008;14:361-368 |
| 30 | Fitzgerald LM, Delitto A, Irrgang JJ. Validation of the Clinical Internship Evaluation Tool. *Phys Ther*. 2007;87;844-860. |
| 31 | Flew B, Chipchase L, Lee D, McClelland JA. Feasibility of an online clinical assessment of competence in physiotherapy students. *Physiother Theory Pract*. 2025;41(3):508-521. DOI: 10.1080/09593985.2024.2344024 |
| 32 | Forbes R, Mandrusiak A. Development and reliability testing of a patient education performance tool for physical therapy students. *Journal of Physical Therapy Education*. 2019;33(1):64-69. DOI: 10.1097/JTE.0000000000000074 |
| 33 | Fu W. Development of an innovative tool to assess student physical therapists' clinical reasoning competency. *Journal of Physical Therapy Education*. 2015;29(4):14-26. |
| 34 | Fuentes-Cimma J, Fuentes-López E, Isbej Espósito L, et al. Utility analysis of an adapted Mini-CEX WebApp for clinical practice assessment in physiotherapy undergraduate students. *Frontiers in Education.* 2023;8:943709. doi: 10.3389/feduc.2023.943709 |
| 35 | Fulton T, Myatt K, Kirwan GW, Clark CR, Dalton M. Rating of physiotherapy student clinical performance in a paediatric setting: Are assessors consistent in their rating of a simulated clinical student performance? *BMC Med Educ*. 2023;23(1):280. doi: 10.1186/s12909-023-04149-9 |
| 36 | Furze J, Gale JR, Black L, Cochran TM, Jensen GM. Clinical reasoning: Development of a grading rubric for student assessment. *Journal of Physical Therapy Education*. 2015;29(3):34-45. |
| 37 | Garcia-Ros R, Ruescas-Nicolau MA, Cezón-Serrano N, et al. Students' Perceptions of instructional rubrics in neurological physical therapy and their effects on students' engagement and course satisfaction. *Int J Environ Res Public* *Health.* 2021;18(9):4957. doi: 10.3390/ijerph18094957 |
| 38 | Garcia-Ros R, Ruescas-Nicolau MA, Cezón-Serrano N, Flor-Rufino C, Martin-Valenzuela CS, Sánchez-Sánchez ML. Improving assessment of procedural skills in health sciences education: a validation study of a rubrics system in neurophysiotherapy. *BMC Psychol.* 2024;12(1):147. doi: 10.1186/s40359-024-01643-7 |
| 39 | Gittinger FP, Lemos M, Neumann JL, et al. Interrater reliability in the assessment of physiotherapy students. *BMC Med Educ*. 2022;22(1):186. doi: 10.1186/s12909-022-03231-y |
| 40 | Gomez Miranda LA, Amigo Reyes T, de la Barra Ortiz HA. Validating an objective structured clinical examination to enhance assessment of clinical skills in physical therapy students. *Journal of Advanced Pharmacy Education and* *Research*. 2024;14(2):16-26. doi: 10.51847/c2DlK9b9pQ |
| 41 | Gorman SL, Lazaro R, Fairchild J, Kennedy B. Development and implementation of an objective structured clinical examination (OSCE) in neuromuscular physical therapy. *Journal of Physical Therapy Education*. 2010;24(3):62-68. |
| 42 | Greene R, Rogers GL. Justifying core faculty assessment of students' clinical performance using cognitive flexibility theory: A case example. *Internet Journal of Allied Health Sciences & Practice*. 2006;4(3):1-5. |
| 43 | Hayward LM, Blackmer B, Markowski A. Standardized patients and communities of practice: A realistic strategy for integrating the core values in a physical therapist education program. *Journal of Physical Therapy Education*. 2006;20(2):29-37. |
| 44 | Hayward LM, Blackmer B. A model for teaching and assessing core values development in Doctor of Physical Therapy students. *Journal of Physical Therapy Education*. 2010;24(3):16-26. |
| 45 | Heijne A, Nordgren B, Hagströmer M, Friden C. Assessment by portfolio in a physiotherapy programme. *Advances in Physiotherapy.* 2012;14:38-46. |
| 46 | Hrachovy J, Clopton N, Baggett K, Garber T, Cantwell J, Schreiber J. Use of the Blue MACS: Acceptance by clinical instructors and self-reports of adherence. *Physical Therapy*. 2000;80:652-661. |
| 47 | Hu J, Jones AYM, Zhou X, et al. Acceptance of the 'Assessment of Physiotherapy Practice (Chinese)' as a standardised evaluation of professional competency in Chinese physiotherapy students: An observational study. *BMC Medical Education.* 2020;20:108. |
| 48 | Johnston TE. Assessment of medical screening and clinical reasoning skills by physical therapy students in a simulated patient encounter. *Internet Journal of Allied Health Sciences and Practice.* 2018;16(2):Article 10. |
| 49 | Jones A, Mandrusiak A, Judd B, Gordon C, Alison J. Investigating a physiotherapy clinical simulation assessment tool using the Delphi approach. Internet Journal of Allied Health Sciences and Practice. 2017;15(3):Article 3. |
| 50 | Jones M, van Kessel G, Swisher L, Beckstead J, Edwards I. Cognitive maps and the structure of observed learning outcome assessment of physiotherapy students' ethical reasoning knowledge. *Assessment & Evaluation in Higher Education.* 2014;39(1):1-20. |
| 51 | Joseph C, Frantz J, Hendricks C, Smith M. Evaluation of a new clinical performance assessment tool: A reliability study. *South African Journal of Physiotherapy.* 2012;68(3):15-19. |
| 52 | Joseph C, Hendricks C, Frantz J. Exploring the key performance areas and assessment criteria for the evaluation of students' clinical performance: A Delphi study. *South African Journal of Physiotherapy.* 2011;67(2):9-15. |
| 53 | Judd BK, Scanlan JN, Alison JA, Waters D, Gordon CJ. The validity of a professional competence tool for physiotherapy students in simulation-based clinical education: A Rasch analysis. *BMC Medical Education.* 2016;16:196. doi: 10.1186/s12909-016-0718-x |
| 54 | Kanada Y, Sakurai H, Sugiura Y, Motoya I, Tomita M, Sawa S, Teranishi T, Okanisi T. Standardizing the assessment of the clinical abilities of physical therapists and occupational therapists using OSCE. *Journal of Physical Therapy Science*. 2012;24(10):985-989. |
| 55 | Kanada Y, Sakurai H, Sugiura Y. Difficulty levels of OSCE items related to examination and measurement skills. *Journal of Physical Therapy Science.* 2015;27(3):715-718. |
| 56 | Kern BP, Mickelson JM. The development and use of an evaluation instrument for clinical education. *Physical Therapy.* 1971;51(5):540-546. |
| 57 | Kinirons SA, Reddin VM. Early identification of students at risk for academic difficulty in a Doctor of Physical Therapy program. *Journal of Allied Health.* 2023;52(1):e9-e15. |
| 58 | Kirwan GW, Clark CR, Dalton M. Rating of physiotherapy student clinical performance: Is it possible to gain assessor consistency? *BMC Medical Education.* 2019:19:32. doi.org/10.1186/s12909-019-1459-4 |
| 59 | Kojich L, Miller SA, Axman K, Eacret T, Koontz JA, Smith C. Evaluating clinical reasoning in first year DPT students using a script concordance test. *BMC Med Educ*. 2024;24(1):329. doi: 10.1186/s12909-024-05281-w |
| 60 | Kosmahl EM. Factors related to physical therapist license examination scores. *Journal of Physical Therapy Education.* 2005;19(2):52-56. |
| 61 | Kulkarni M, Sinha R, Sinha S, Mahajan A. Perception of undergraduate physiotherapy students toward objectively structured practical examination checklist as a tool for assessment and learning active cycle of breathing techniques skill: A cross-sectional descriptive study. *Indian Journal of Physical Therapy & Research.* 2023;5(2):182-186. |
| 62 | Ladyshewsky R, Baker R, Jones M, Nelson L. Evaluating clinical performance in physical therapy with simulated patients. *Journal of Physical Therapy Education.* 2000;14(1):31-37. |
| 63 | Ladyshewsky R, Baker R, Jones M, Nelson L. Reliability and validity of an extended simulated patient case: A tool for evaluation and research in physiotherapy. *Physiotherapy Theory and Practice*. 2000;16:15-25. |
| 64 | Lewis LK, Stiller K, Hardy F. A clinical assessment tool used for physiotherapy students - Is it reliable? *Physiother Theory Pract.* 2008;24(2):121-134. |
| 65 | Lo K, Osadnik C, Leonard M, Maloney S. Differences in student and clinician perceptions of clinical competency in undergraduate physiotherapy. *New Zealand Journal of Physiotherapy.* 2015;43(1):11-15. doi: 10.15619/NZJPI43.1.02 |
| 66 | Loomis J. Evaluating clinical competence of physical therapy students. Part 1: The development of an instrument. *Physiotherapy Canada.* 1985;37(2):83-89. |
| 67 | Loomis J. Evaluating clinical competence of physical therapy students. Part 2: Assessing the reliability, validity and usability of a new instrument. *Physiotherapy Canada.* 1985;37(2):91-98. |
| 68 | Luedtke-Hoffmann K, Dillon L, Utsey C, Tomaka J. Is there a relationship between performance during physical therapist clinical education and scores on the National Physical Therapy Examination (NPTE)? *Journal of Physical Therapy Education.* 2012;26(2):41-49. |
| 69 | Lysaght C, Lin CC, Stokes CK, Kim YJ, Murphy L, Hopkins P, Radloff J. Interprofessional collaborative care skills and behaviors: Perception differences between allied health students and an independent observer. *J Allied Health*. 2022;51(4):e77-e84. |
| 70 | Martiáñez-Ramírez NL, Pineda-Galán C, Rodríguez-Bailón M, Romero-Galisteo RP. Competence assessment rubric in the Physiotherapy Practicum. *PLoS One.* 2022;17(2):e0264120. doi: 10.1371/journal.pone.0264120 |
| 71 | Mays MJ. Reliability of a method of evaluating the clinical performance of a physical therapy student. *Physical Therapy.* 1973;53(12):1298-1306. |
| 72 | McDevitt A, Rapport MJ, Jensen G, Furze J. Utilization of the Clinical Reasoning Assessment Tool across a physical therapy curriculum: Application for teaching, learning, and assessment. *Journal of Physical Therapy Education*. 2019;33(4):335-342. |
| 73 | McDevitt AD, Rapport MJD, Rodriguez JD, Miller M. Faculty perceptions on use of the Clinical Reasoning Assessment Tool to support learning in physical therapist students: A qualitative study. *Journal of Physical Therapy Education*. 2022;36(1):57-64. doi: 10.1097/JTE.0000000000000207 |
| 74 | Meiners KM, Rush DK. Clinical performance and admission variables as predictors of passage of the National Physical Therapy Examination. *Journal of Allied Health*. 2017;46(3):164-170. |
| 75 | Meldrum D, Lydon A, Loughnane M, Geary F, Shanley L, Sayers K, Shinnick E, Filan D. Assessment of undergraduate physiotherapist clinical performance: investigation of educator inter-rater reliability. *Physiotherapy*. 2008;94(3):212-219. doi:10.1016/j.physio.2008.03.003 |
| 76 | Miller AH, Cummings N, Tomlinson J. Measurement error and detectable change for the modified Fresno Test in first-year entry-level physical therapy students. *J Allied Health.* 2013;42(3):169-74. |
| 77 | Mori B, Brooks D, Norman KE, Herold J, Beaton DE. Development of the Canadian Physiotherapy Assessment of Clinical Performance: A new tool to assess physiotherapy students' performance in clinical education. *Physiother* *Can*. 2015;67(3):281-9. doi:10.3138/ptc.2014-29E |
| 78 | Mori B, Daly A, Norman KE, Wojkowski S. The development of the Canadian Physiotherapy Assessment of Clinical Performance (ACP) 2.0 - Alignment with the 2017 Competency Profile. *Physiother Can*. 2024;76(1):111-120. doi:10.3138/ptc-2021-0077 |
| 79 | Mori B, Norman KE, Brooks D, Herold J, Beaton DE. Canadian Physiotherapy Assessment of Clinical Performance: Face and content validity. *Physiother Can.* 2016;68(1):64-72. doi:10.3138/ptc.2015-35E |
| 80 | Mori B, Norman KE, Brooks D, Herold J, Beaton DE. Evidence of reliability, validity, and practicality for the Canadian Physiotherapy Assessment of Clinical Performance. *Physiother Can*. 2016;68(2):156-169. doi: 10.3138/ptc.2014-43E |
| 81 | Morris J. Audit of use of a common undergraduate physiotherapy clinical assessment form. *International Journal of Therapy & Rehabilitation.* 2006;13(9):407-413. |
| 82 | Muhamad Z; Ramli A; Amat S. Validity and reliability of the Clinical Competency Evaluation Instrument for use among physiotherapy students: Pilot study. *Sultan Qaboos Univ Med J*. 2015;15(2):e266-74. |
| 83 | Murphy S, Dalton M, Dawes D. Assessing physical therapy students' performance during clinical practice. *Physiother Can.* 2014;66(2):169-76. doi:10.3138/ptc.2013-26 |
| 84 | Naylor S, Norris M, Williams A. Does ethnicity, gender or age of physiotherapy students affect performance in the final clinical placements? An exploratory study. *Physiotherapy*. 2014;100(1):9-13. doi: 10.1016/j.physio.2013.05.004 |
| 85 | Nesbit KC, Fitzsimmons A. Grappling with professionalism: A developmental approach to a dynamic concept. *Journal of Physical Therapy Education.* 2021;35(2):103-112. doi: 10.1097/JTE.0000000000000174 |
| 86 | Norman K, Booth R. Observations and performances "with distinction" by physical therapy students in clinical education: Analysis of checkboxes on the Physical Therapist Clinical Performance Instrument (PT-CPI) over a 4-year period. *Physiother Can.* 2015;67(1):17-29. |
| 87 | North S, Sharp A. Embracing change in the pursuit of excellence: Transitioning to the Clinical Internship Evaluation Tool for student clinical performance assessment. *Journal of Physical Therapy Education.* 2020;34(4):313-320. DOI: 10.1097/JTE.0000000000000154 |
| 88 | O'Malley E, Scanlon AM, Alpine L, McMahon S. Enabling the feedback process in work-based learning: An evaluation of the 5 minute feedback form. *Assessment & Evaluation in Higher Education.* 2021;46(7):1020-1034. doi: 0.1080/02602938.2020.1842852 |
| 89 | Oldmeadow L. Developing clinical competence: a mastery pathway. *Aust J Physiother.* 1996;42(1):37-44. |
| 90 | Pabian PS, Kay D, Neely L, Whitworth J. An interprofessional education approach to pain management through a standardized patient encounter. *Journal of Interprofessional Education and Practice*. 2022;29:100568. doi: 10.1016/j.xjep.2022.100568 |
| 91 | Panzarella KJ, Manyon AT. A model for integrated assessment of clinical competence. *J Allied Health.* 2007;36(3):157-64. |
| 92 | Panzarella KJ, Manyon AT. Using the Integrated Standardized Patient Examination to assess clinical competence in physical therapist students. *Journal of Physical Therapy Education.* 2008;22(3):24-32. |
| 93 | Panzarella KJ. Beginning with the end in mind: Evaluating outcomes of cultural competence instruction in a doctor of physical therapy programme. *Disabil Rehabil.* 2009;31(14):1144-1152. DOI: 10.1080/09638280902773745 |
| 94 | Pata R, O'Sullivan S, Peterson D, Superchi L, Feinn, R. Simulation-based Cardiopulmonary Examination Skills Checklist: Assessment of inter-rater reliability. *Cardiopulmonary Physical Therapy Journal.* 2024;35(3):113-123. doi: 10.1097/CPT.0000000000000251 |
| 95 | Pechak C, Dillon L, Umucu E. Improving patient-provider communication: Evolution of a tool to assess physical therapist students' Spanish-language proficiency. *Health Commun*. 2019;34(12):1433-1440. doi: 10.1080/10410236.2018.1495161 |
| 96 | Pérez-Guillén S, Carrasco-Uribarren A, Celis CL, González-Rueda V, Rodríguez-Rubio PR, Cabanillas-Barea S. Students' perceptions, engagement and satisfaction with the use of an e-rubric for the assessment of manual skills in physiotherapy. *BMC Med Educ*. 2022;22(1):623. doi: 10.1186/s12909-022-03651-w |
| 97 | Plack MM, Driscoll M, Blissett S, McKenna R, Plack TP. A method for assessing reflective journal writing. *J Allied Health.* 2005;34(4):199-208. |
| 98 | Proctor PL, Dal Bello-Haas VP, McQuarrie AM, Sheppard MS, Scudds RJ. Scoring of the physical therapist clinical performance instrument (PT-CPI): analysis of 7 years of use. *Physiother Can.* 2010;62(2):147-54. DOI:10.3138/physio.62.2.147 |
| 99 | Reubenson A, Ng L, Gucciardi DF. The Assessment of Physiotherapy Practice tool provides informative assessments of clinical and professional dimensions of student performance in undergraduate placements: A longitudinal validity and reliability study. *J Physiother.* 2020;66(2):113-119. doi: 10.1016/j.jphys.2020.03.009 |
| 100 | Rheault W, Coulson E. Use of the Rasch model in the development of a clinical competence scale. *Journal of Physical Therapy Education.* 1991;5(1):10-13. |
| 101 | Riolo L. Reliability of assessing psychomotor tasks in physical therapy curricula. *Journal of Physical Therapy Education.* 1997;11(1):36-39. |
| 102 | Roach KE, Frost JS, Francis NJ, Giles S, Nordrum JT, Delitto A. Validation of the Revised Physical Therapist Clinical Performance Instrument (PT CPI): Version 2006. *Phys Ther.* 2012;92(3):416-28. |
| 103 | Roth HR, Holland EE, Goh L, Wong E, McGaghie WC, Tappan RS. Systematic development and validity evidence for a checklist to assess bed mobility skills among physical therapy students. *Journal of Allied Health.* 2024;53(2):122-129. |
| 104 | Ryall T, Preston E, Bissett B. Can classroom-based Peer Patient simulation predict real-life clinical performance in physiotherapy students? *Heliyon*. 2025;11(6):e43027. doi: 10.1016/j.heliyon.2025.e43027 |
| 105 | Sakurai H, Kanada Y, Sugiura Y, Motoya I, Yamada M, Tomita M, Naka T, Teranishi T, Tanabe S, Okanisi T. Standardization of clinical competency evaluation in the education of physical therapists and occupational therapists - Establishment of an OSCE compliant education system. *Journal of Physical Therapy Science.* 2013;25(1):101-107. |
| 106 | Sattelmayer KM, Jagadamma KC, Sattelmayer F, Hilfiker R, Baer G. The assessment of procedural skills in physiotherapy education: A measurement study using the Rasch model. *Arch Physiother*. 2020;10():9. |
| 107 | Schreiber J, Gagnon K, Kendall E, LaForme Fiss A, Rapport MJ, Wynarczuk KD. Development of a grading rubric to assess learning in pediatric physical therapy education. *Pediatr Phys Ther.* 2020;32(1):70-79. doi: 10.1097/PEP.0000000000000667 |
| 108 | Schwartz D, Jacob T. Establishing the reliability of a tool for assessing Israeli physical therapy students' clinical performance. Journal of Physical Therapy Education. 2019;33(3):243-248. doi: 10.1097/JTE.0000000000000093 |
| 109 | Seif GA, Kraft SV, Bowden MG, Boissonnault JS. Intra-rater reliability of the ECHOWS Tool for real-time assessment of physical therapy student interviewing skills: A pilot study. *Health Professions Education.* 2019;5(2):146-151. doi: 10.1016/j.hpe.2018.05.001 |
| 110 | Seymour CJ, Dybel GJ. Developing skillful clinical decision making: Evaluation of two classroom teaching strategies. *Journal of Physical Therapy Education.* 1996;10(2):77-81. |
| 111 | Silva AM, Costa LCM, Comper ML, Padula RS. Cross-cultural adaptation and reproducibility of the Brazilian-Portuguese version of the modified FRESNO Test to evaluate the competence in evidence based practice by physical therapists. *Brazilian Journal of Physical Therapy.* 2016;20(1):26-47. doi: 10.1590/bjpt-rbf.2014.0140 |
| 112 | Silva CCBM, Lunardi AC, Mendes FAR, Souza FFP, Carvalho CRF. Objective structured clinical evaluation as an assessment method for undergraduate chest physical therapy students: a cross-sectional study. *Brazilian Journal of Physical Therapy.* 2011;15(6):481-486. |
| 113 | Sliwinski MM, Schultze K, Hansen RL, Malta S, Babyar SR. Clinical performance expectations: A preliminary study comparing physical therapist students, clinical instructors, and academic faculty. *Journal of Physical Therapy Education*. 2004;18(1):50-57. |
| 114 | Smeets HWH, Delnoij LEC, Sluijsmans DMA, Moser A, van Merrienboer JJG. From individual to interprofessional: Characteristics of assessment tasks to assess interprofessional collaboration in healthcare education. *J Interprof Care*. 2024;38(5):907-917. DOI: 10.1080/13561820.2024.2381058 |
| 115 | Smeets HWH; Delnoij LEC, Sluijsmans DMA, Moser A, van Merrienboer JJG. The balancing act of assessment validity in interprofessional healthcare education: A qualitative evaluation study. *Teaching & Learning in Medicine*. 2025;37(1):99-112. DOI: 10.1080/10401334.2023.2280855 |
| 116 | Stevens K, Henderson H, Hawthorne K, Carlson J. A comparison of methods for setting passing scores in standardized simulated patient experiences in physical therapist education. *Journal of Physical Therapy Education.* 2013;27(3):78-81. |
| 117 | Stickley LA. Content validity of a clinical education performance tool: the Physical Therapist Manual for the Assessment of Clinical Skills. *Journal of Allied Health.* 2005;34(1):24-30. |
| 118 | Straube D, Campbell SK. Rater discrimination using the visual analog scale of the Physical Therapist Clinical Performance Instrument. *Journal of Physical Therapy Education.* 2003;17(1):33-38. |
| 119 | Swift M, Spake E, Gajewski BJ. The reliability of a musculoskeletal objective structured clinical examination in a professional physical therapist program. *Journal of Physical Therapy Education.* 2013;27(2):41-48. |
| 120 | Swift M, Spake E, Kohia, M. Examiner fatigue and ability to concentrate in objective structured clinical examinations for physical therapist students. *Journal of Allied Health.* 2016;45(1):62-70. |
| 121 | Tappan RS, Hedman LD, López-Rosado R, Roth HR. Checklist-style rubric development for practical examination of clinical skills in entry-level physical therapist education. *J Allied Health.* 2020;49(3):202-207. |
| 122 | Task Force for the Development of Student Clinical Performance Instruments. The development and testing of APTA Clinical Performance Instruments. *Phys Ther*. 2002;82(4):329-353. |
| 123 | Thieman TJ, Weddle ML, Moore MA. Predicting academic, clinical, and licensure examination performance in a professional (entry-level) master's degree program in physical therapy. *Journal of Physical Therapy Education.* 2003;17(2):32-37. |
| 124 | Tilson JK. Validation of the modified Fresno Test: Assessing physical therapists' evidence based practice knowledge and skills. *BMC Medical Education.* 2010;10(1):38. |
| 125 | Torres-Narváez MR; Vargas-Pinilla OC; Rodríguez-Grande EIVali. dity and reproducibility of a tool for assessing clinical competencies in physical therapy students. *BMC Med Educ*. 2018;18(1):280. doi: 0.1186/s12909-018-1377-x |
| 126 | Tunney N, Perlow E. Student and examiner perceptions of an innovative model for assessment of neuromuscular clinical competence in a professional physical therapist education program. *Journal of Physical Therapy Education.* 2017;31(3):91-99. |
| 127 | Turbow DJ, Evener J. Norming a VALUE rubric to assess graduate information literacy skills. *J Med Libr Assoc.* 2016;104(3):209-14. doi: 10.3163/1536-5050.104.3.005 |
| 128 | Vendrely A, Carter R. The influence of training on the rating of physical therapist student performance in the clinical setting. *J Allied Health.* 2004;33(1):62-69. |
| 129 | Weeks BK, Laakso L. Using debates as assessment in a physiotherapy capstone course: A case example. *Journal of University Teaching and Learning Practice.* 2016;13(3):8. doi: 10.53761/1.13.3.8 |
| 130 | Wessel J, Williams R, Finch E, Gémus M. Reliability and validity of an objective structured clinical examination for physical therapy students. *J Allied Health*. 2003;32(4):266-9. |
| 131 | Wetherbee E, Dupre AM, Feinn RS, Roush S. Relationship between narrative comments and ratings for entry-level performance on the Clinical Performance Instrument: A call to rethink the Clinical Performance Instrument. *Journal of Physical Therapy Education.* 2018;32(4):333-343. DOI: 10.1097/JTE.0000000000000060 |
| 132 | White LW, Jordan KE, McDermott H. Assessment of student readiness for clinical education in mixed-mode curriculum delivery: A case study. *Quality Assurance in Education: An International Perspective*. 2023;31(1):151-166. |
| 133 | Williams R, Sanford J, Stratford PW, Newman A. Grading written essays: A reliability study. *Physical Therapy*. 1991;71(9):679-686. |
| 134 | Wolden B, Wolden M, Furze J, McDevitt A. Advancing consistency in education: A reliability analysis of the Clinical Reasoning Assessment Tool. *J Phys Ther Educ*. 2024;online ahead of print. DOI: 10.1097/JTE.0000000000000365 |
| 135 | Wolden M, Drevyn E, Flom-Meland C, Gusman, Lori N. Evaluation and modification of the Physical Therapist Clinical Performance Instrument. *Journal of Physical Therapy Education.* 2021;35(2):85-94. DOI: 10.1097/JTE.0000000000000180 |
| 136 | Wong CK, Blissett S. Assessing performance in the area of cultural competence: An analysis of reflective writing. *Journal of Physical Therapy Education*. 2007;21(1):40-47. |
| 137 | Yeldon J, Wilson R, Laferriére J, et al. Let's talk about the talk: Exploring the experience of discussing student performance at the mid- and final points of the clinical internship. *Physiother Can.* 2018;70(3):240-248. doi:10.3138/ptc.2016-96 |
| 138 | Zhang C, Miller C, Volkman K, Meza J, Jones K. Evaluation of the team performance observation tool with targeted behavioral markers in simulation-based interprofessional education. *J Interprof Care.* 2015;29(3):202-208. doi: 10.3109/13561820.2014.982789 |
| 139 | Zhang G, Fenderson BA, Schmidt RR, Veloski JJ. Equivalence of students' scores on timed and untimed anatomy practical examinations. *Anat Sci Educ.* 2013;6(5):281-5. |
