# Supplementary material for: Educational assessments in entry-level physical therapy education: a scoping review
Source: BMC Med Educ. 2026 Mar 5;26:592. doi: 10.1186/s12909-026-08927-z (PMC13069796; doi:10.1186/s12909-026-08927-z)
Supplement: Supplementary file 3 — Supplementary Material 3. Title and Description: Scoping Review Supplement 3 Frequency of Data Elements. Validity evidence data elements and prevalence for each of the five sources of validity evidence. Five tables list the types of validity evidence data elements identified and the prevalence of each data element for each type of validity evidence: content, response process, internal structure, relations with other variables, consequences. [file 12909_2026_8927_MOESM3_ESM.docx]

Supplement 3a: Validity evidence data elements and prevalence: content evidence

| **Data Element** | **Data Element Definition (adapted from Cook 2014**^4^**)** | **Prevalence by Study,**  **No. (%)**  **N = 139** | **Prevalence by Assessment,**  **No. (%)**  **N=92** |
| --- | --- | --- | --- |
| **Any** |  | 78 (56.1%) | 64 (70.0%) |
| **Expert Panel** | Group consensus or expert review, Including Delphi review, survey, and interview methods. | 58 (41.7%) | 48 (52.2%) |
| **Adaptation from Previous Instrument** | Assessment based on or modified from a previous instrument | 11 (7.9%) | 11 (12.0%) |
| **Pilot Testing and Revision** | Pilot testing and revision of assessment for iterative development | 18 (12.9%) | 15 (16.3%) |
| **Guidelines** | Use of clinical, educational, or other high quality evidence guidelines to determine assessment content | 11 (7.9%) | 11 (12.0%) |
| **Scoring Framework** | Scoring development based on theory, framework, or test blueprint | 6 (4.3%) | 6 (6.5%) |
| **Test Blueprint** | Use of framework, learning objectives, or other test blueprint to systematically determine assessment content | 11 (7.9%) | 11 (12.0%) |

Supplement 3b: Validity evidence data elements and prevalence: response process evidence

| **Data Element** | **Data Element Definition (adapted from Cook 2014**^4^**)** | **Prevalence by Study,**  **No. (%)**  **N = 139** | **Prevalence by Assessment,**  **No. (%)**  **N=92** |
| --- | --- | --- | --- |
| **Any** |  | 43 (30.9%) | 32 (34.8%) |
| **Analysis of Assessment Data** | Analysis of rater data/ratings from the assessment, including omissions, disagreements, or errors | 8 (5.8%) | 5 (5.4%) |
| **Test Security** | Evaluation of systems to protect exam integrity and security | 0 (0.0%) | 0 (0.0%) |
| **Evaluation of data collection** | Evaluation of issues related to data collection, e.g., video capture problems, standardizing patient presentation (SP training) | 5 (3.6%) | 5 (5.4%) |
| **Effect of Rater Training** | Evaluation of the effect of rater training | 11 (7.9%) | 10 (10.9%) |
| **Rater Thought Processes** | Evaluation of raters’ internal cognitive processes during interaction with the assessment, includes methods such as the think aloud protocol and cognitive interviewing. | 4 (2.9%) | 4 (4.3%) |
| **Evaluation of End User Experiences** | Evaluation of rater and examinee experiences and perceptions of the examination process after the assessment is completed, includes methods such as survey or interview | 21 (15.1%) | 18 (20.0%) |

Supplement 3c: Validity evidence data elements and prevalence: internal structure evidence

| **Data Element** | **Data Element Definition (adapted from Cook 2014**^4^**)** | **Prevalence by Study,**  **No. (%)**  **N = 139** | **Prevalence by Assessment,**  **No. (%)**  **N=92** |
| --- | --- | --- | --- |
| **Any** |  | 70 (50.4%) | 49 (53.2%) |
| **Reliability, Any** | Reproducibility of scores | 59 (42.4%) | 44 (47.8%) |
| **Internal Consistency** | Reliability across test items using classical test theory | 30 (21.6%) | 23 (25.0%) |
| **Intrarater Reliability** | Reliability within raters, using classical test theory | 10 (7.2%) | 6 (6.5%) |
| **Interrater Reliability** | Reliability across raters, using classical test theory | 42 (30.2%) | 33 (35.9%) |
| **Test-Retest Reliability** | Reliability across different test versions, using classical test theory | 6 (4.3%) | 5 (5.4%) |
| **Other Reliability** | Reliability testing using Generalizability Theory or Analysis of Variance to examine reliability across facets | 2 (1.4%) | 2 (2.2%) |
| **Analysis of Data Distribution** | Evaluation of data distribution | 3 (2.2%) | 2 (2.2%) |
| **Empiric Scoring** | Scoring method analysis using empiric data, including Rasch analysis | 3 (2.2%) | 3 (3.3%) |
| **Item Analysis** | Evaluation of item performance, including inter-item correlation and item discrimination | 10 (7.2%) | 9 (9.8%) |
| **Factor Analysis** | Exploratory or confirmatory factor analysis | 16 (11.5%) | 9 (9.8%) |
| **Other** |  | 1 (0.7%) | 1 (1.1%) |

Supplement 3d: Validity evidence data elements and prevalence: relations with other variables evidence

| **Data Element** | **Data Element Definition (adapted from Cook 2014**^4^**)** | **Prevalence by Study,**  **No. (%)**  **N = 139** | **Prevalence by Assessment,**  **No. (%)**  **N=92** |
| --- | --- | --- | --- |
| **Any** |  | 47 (33.8%) | 37 (40.2%) |
| **Learner Characteristic** | Association with a learner characteristic, e.g., level of training | 28 (20.1%) | 22 (23.9%) |
| **Separate Measure, Concurrent** | Association with a separate measure, with the variables measured within 1 month of each other | 17 (12.2%) | 14 (15.2%) |
| **Separate Measure, Delayed** | Association with a separate measure, with the variables measured ≥1 month apart | 14 (10.1%) | 9 (9.8%) |

Supplement 3e: Validity evidence data elements and prevalence: consequences evidence

| **Data Element** | **Data Element Definition (adapted from Cook 2014**^4^**)** | **Prevalence by Study,**  **No. (%)**  **N = 139** | **Prevalence by Assessment,**  **No. (%)**  **N=92** |
| --- | --- | --- | --- |
| **Any** |  | 36 (25.9%) | 31 (33.7%) |
| **Rigorous pass/fail cut score, established approach** | Pass/fail cut-off set using an established standard setting method, e.g., Angoff method, borderline group method, Hofstee method) | 1 (0.7%) | 1 (1.1%) |
| **Rigorous pass/fail cut score, novel approach** | Pass/fail cut-off set using a novel, but rigorous approach | 1 (0.7%) | 1 (1.1%) |
| **Evaluation of pass rate** | Evaluation of actual test results, including the pass rate | 13 (8.6%) | 12 (13.0%) |
| **Anticipated Impact** | Anticipated impact of the assessment on students, faculty, patients, or others; may be positive or negative | 16 (11.5%) | 16 (17.4%) |
| **Unanticipated Impact** | Unanticipated impact of the assessment on students, faculty, patients, or others; may be positive or negative; includes evaluation of assessment bias, such as differential item functioning | 13 (9.4%) | 11 (12.0%) |
