# Supplementary material for: Educational assessments in entry-level physical therapy education: a scoping review
Source: BMC Med Educ. 2026 Mar 5;26:592. doi: 10.1186/s12909-026-08927-z (PMC13069796; doi:10.1186/s12909-026-08927-z)
Supplement: Supplementary file 4 — Supplementary Material 4. Title and Description: Scoping Review Supplement 4 List of Articles with Assessment and Validity Evidence Characteristics. Included studies with assessment and validity evidence characteristics. Evidence ratings range 0-3 and are listed for each source of validity evidence. See Table 2 for validity evidence rating anchors. Reference list is included at the end of this document. [file 12909_2026_8927_MOESM4_ESM.docx]

Additional File 4:

List of included papers with assessment and validity evidence characteristics. Evidence ratings range 0-3 and are listed for each source of validity evidence. See Table 2 for validity evidence rating anchors. Reference list is included at the end of this document.

Abbreviations: PT = physical therapist; PTA = physical therapist assistant; IPE = interprofessional education (including PTs and/or PTAs); ≥1 = more than one discipline (including PTs and/or PTAs) included in the study, but not IPE; Rel. w/ Other Var. = Relations with Other Variables

|  |  |  |  | **Evidence Rating** | | | | |  |
| --- | --- | --- | --- | --- | --- | --- | --- | --- | --- |
| **Target Construct(s)** | **Assessment Name** | **Assessment Format(s)** | **Discipline** | **Content** | **Response Process** | **Internal Structure** | **Rel. w/ Other Var.** | **Consequences** | **Reference** |
| Anatomy Knowledge | N/A | Written - Multiple Choice; Written - Other | PT | 0 | 0 | 0 | 2 | 0 | Kinirons SA (2023)^1^ |
| Anatomy Knowledge | N/A | Written - Multiple Choice | PT | 0 | 2 | 1 | 0 | 0 | Zhang G (2013)^2^ |
| Anatomy Knowledge | Oral Anatomy Laboratory Examination | Oral | PT | 2 | 0 | 0 | 0 | 1 | Fabrizio PA (2013)^3^ |
| Anatomy Knowledge | Remediation Computer Practical Examination | Written - Multiple Choice; Written – Other (short answer) | ≥1 | 0 | 0 | 0 | 0 | 1 | Daly FJ (2010)^4^ |
| Bed Mobility Skills | Bed Mobility Skills Checklist | Performance - OSCE/Simulation | PT | 3 | 0 | 1 | 0 | 0 | Roth HR (2024)^5^ |
| Cardiopulmonary Examination Skills | Cardiopulmonary Examination Skills Checklist | Performance - OSCE/Simulation | PT | 2 | 0 | 1 | 0 | 0 | Pata R (2024)^6^ |
| Cardiopulmonary Examination Skills | N/A | Written - Essay; Performance - OSCE/Simulation | PT | 0 | 0 | 0 | 0 | 1 | Balaraman T (2021)^7^ |
| Cardiopulmonary Intervention Skills | Active Cycle of Breathing Techniques Objectively Structured Practical Examination (OSPE) | Performance - OSCE/Simulation | PT | 1 | 2 | 0 | 0 | 1 | Kulkarni M (2023)^8^ |
| Cardiopulmonary Intervention Skills | N/A | Performance - OSCE/Simulation | PT | 0 | 0 | 1 | 1 | 0 | Silva CCBM (2011)^9^ |
| Clinical Competence | Assessment of Physiotherapy Practice (APP) | Performance - Workplace-Based | PT | 2 | 0 | 3 | 0 | 2 | Dalton M (2011)^10^ |
| Clinical Competence | Assessment of Physiotherapy Practice (APP) | Performance - Workplace-Based | PT | 0 | 0 | 2 | 0 | 0 | Dalton M (2012)^11^ |
| Clinical Competence | Assessment of Physiotherapy Practice (APP) | Performance - Workplace-Based | PT | 0 | 0 | 1 | 0 | 0 | Fulton T (2023)^12^ |
| Clinical Competence | Assessment of Physiotherapy Practice (APP) | Performance - OSCE/Simulation | PT | 2 | 0 | 0 | 0 | 0 | Jones A (2017)^13^ |
| Clinical Competence | Assessment of Physiotherapy Practice (APP) | Performance - OSCE/Simulation | PT | 2 | 0 | 2 | 0 | 2 | Judd BK (2016)^14^ |
| Clinical Competence | Assessment of Physiotherapy Practice (APP) | Performance - Workplace-Based | PT | 0 | 1 | 1 | 0 | 0 | Kirwan GW (2019)^15^ |
| Clinical Competence | Assessment of Physiotherapy Practice (APP) | Performance - Workplace-Based | PT | 0 | 0 | 0 | 1 | 0 | Lo K (2015)^16^ |
| Clinical Competence | Assessment of Physiotherapy Practice (APP) | Performance - Workplace-Based | PT | 0 | 2 | 0 | 1 | 2 | Murphy S (2014)^17^ |
| Clinical Competence | Assessment of Physiotherapy Practice (APP) | Performance - Workplace-Based | PT | 0 | 0 | 3 | 0 | 0 | Reubenson A (2020)^18^ |
| Clinical Competence | Assessment of Physiotherapy Practice (APP) - Chinese version | Performance - Workplace-Based | PT | 2 | 2 | 0 | 0 | 0 | Hu J (2020)^19^ |
| Clinical Competence | Assessment of Physiotherapy Practice (APP) - Hebrew version | Performance - Workplace-Based | PT | 2 | 0 | 2 | 1 | 0 | Schwartz D (2019)^20^ |
| Clinical Competence | Assessment of Physiotherapy Practice (APP) - Turkish version | Performance - Workplace-Based | PT | 2 | 0 | 3 | 0 | 1 | Çelik H (2024)^21^ |
| Clinical Competence | Clinical Competency Evaluation Instrument (CCEVI) | Performance - Workplace-Based | PT | 2 | 0 | 3 | 0 | 0 | Muhamad Z (2015)^22^ |
| Clinical Competence | Common Physiotherapy Clinical Assessment Form | Performance - Workplace-Based | PT | 0 | 3 | 0 | 1 | 0 | Morris J (2006)^23^ |
| Clinical Competence | Evaluation of Clinical Competence (ECC) | Performance - Workplace-Based | PT | 3 | 0 | 0 | 0 | 0 | Loomis J (1985)^24^ |
| Clinical Competence | Evaluation of Clinical Competence (ECC) | Performance - Workplace-Based | PT | 2 | 0 | 1 | 2 | 0 | Loomis J (1985)^25^ |
| Clinical Competence | Form B | Performance - Workplace-Based | PT | 2 | 3 | 2 | 0 | 2 | Cross V (2001)^26^ |
| Clinical Competence | Integrated Standardized Patient Examination | Performance - OSCE/Simulation | ≥1 | 1 | 0 | 2 | 0 | 0 | Panzarella KJ (2007)^27^ |
| Clinical Competence | Integrated Standardized Patient Examination | Performance - OSCE/Simulation | PT | 1 | 0 | 3 | 1 | 0 | Panzarella KJ (2008)^28^ |
| Clinical Competence | Measurement Tool for Clinical Competencies in PT (MTCCP) | Performance - Workplace-Based | PT | 3 | 2 | 3 | 0 | 0 | Torres-Narváez MR (2018)^29^ |
| Clinical Competence | N/A | Performance - OSCE/Simulation | PT | 3 | 0 | 0 | 0 | 0 | Costello E (2011)^30^ |
| Clinical Competence | N/A | Performance - OSCE/Simulation | PT | 0 | 0 | 0 | 2 | 0 | Kanada Y (2012)^31^ |
| Clinical Competence | N/A | Performance - OSCE/Simulation | PT | 0 | 0 | 1 | 1 | 0 | Kanada Y (2015)^32^ |
| Clinical Competence | N/A | Performance - Workplace-Based | PT | 1 | 0 | 0 | 0 | 0 | Oldmeadow L (1996)^33^ |
| Clinical Competence | N/A | Performance - Workplace-Based | PT | 1 | 0 | 1 | 0 | 0 | Rheault W (1991)^34^ |
| Clinical Competence | N/A | Performance - OSCE/Simulation | PT | 0 | 0 | 0 | 0 | 1 | Sakurai H (2013)^35^ |
| Clinical Competence | Online Clinical Assessment of Competence | Performance - OSCE/Simulation | PT | 2 | 2 | 0 | 1 | 1 | Flew B (2025)^36^ |
| Clinical Competence | Rubric for Competence Assessment in Clinical Physiotherapy Practices (RECOPC-FIS II) | Performance - Workplace-Based | PT | 0 | 0 | 3 | 1 | 0 | Martiáñez-Ramírez NL (2022)^37^ |
| Clinical Competence, Cultural Competence | Integrated Standardized Patient Examination | Performance - OSCE/Simulation | PT | 0 | 0 | 0 | 0 | 2 | Panzarella KJ (2009)^38^ |
| Clinical Education Readiness | N/A | Performance - OSCE/Simulation | PT | 1 | 0 | 0 | 0 | 1 | White LW (2023)^39^ |
| Clinical Evaluation Skills, Communication | N/A | Performance - OSCE/Simulation | PT | 2 | 2 | 2 | 1 | 0 | Ladyshewsky R (2000)^40^ |
| Clinical Evaluation Skills, Professional Behaviors | N/A | Performance - OSCE/Simulation | PT | 2 | 0 | 0 | 0 | 0 | Hayward LM (2006)^41^ |
| Clinical Evaluation Skills, Professional Behaviors | N/A | Performance - OSCE/Simulation | PT | 2 | 0 | 0 | 0 | 0 | Hayward LM (2010)^42^ |
| Clinical Performance | 5 Minute Feedback Form (5MFF) | Performance - Workplace-Based | PT | 0 | 2 | 0 | 0 | 1 | O'Malley E (2021)^43^ |
| Clinical Performance | Blue MACS | Performance - Workplace-Based | PT | 0 | 1 | 0 | 0 | 0 | Hrachovy J (2000)^44^ |
| Clinical Performance | Canadian Physiotherapy Assessment of Clinical Performance (ACP) | Performance - Workplace-Based | PT | 0 | 2 | 0 | 0 | 0 | Mori B (2016)^45^ |
| Clinical Performance | Canadian Physiotherapy Assessment of Clinical Performance (ACP) | Performance - Workplace-Based | PT | 0 | 2 | 1 | 3 | 0 | Mori B (2016)^46^ |
| Clinical Performance | Canadian Physiotherapy Assessment of Clinical Performance (ACP) | Performance - Workplace-Based | PT | 0 | 0 | 0 | 0 | 1 | Yeldon J (2018)^47^ |
| Clinical Performance | Canadian Physiotherapy Assessment of Clinical Performance (ACP) 2.0 | Performance - Workplace-Based | PT | 3 | 3 | 0 | 0 | 0 | Mori B (2015)^48^ |
| Clinical Performance | Canadian Physiotherapy Assessment of Clinical Performance (ACP) 2.0 | Performance - Workplace-Based | PT | 2 | 1 | 0 | 0 | 0 | Mori B (2024)^49^ |
| Clinical Performance | CAR form | Performance - Workplace-Based | PT | 0 | 0 | 0 | 1 | 2 | Naylor S (2014)^50^ |
| Clinical Performance | Clinical Assessment Form | Performance - Workplace-Based | PT | 2 | 0 | 1 | 0 | 0 | Meldrum D (2008)^51^ |
| Clinical Performance | Clinical Internship Evaluation Tool (CIET) | Performance - Workplace-Based | PT | 2 | 2 | 1 | 0 | 0 | Birkmeier M (2022)^52^ |
| Clinical Performance | Clinical Internship Evaluation Tool (CIET) | Performance - Workplace-Based | PT | 3 | 0 | 2 | 2 | 1 | Fitzgerald LM (2007)^53^ |
| Clinical Performance | Clinical Internship Evaluation Tool (CIET) | Performance - Workplace-Based | PT | 1 | 2 | 0 | 1 | 1 | North S (2020)^54^ |
| Clinical Performance | Clinical Performance Assessment Form | Performance - Workplace-Based | PT | 2 | 0 | 2 | 0 | 0 | Joseph C (2012)^55^ |
| Clinical Performance | Clinical Performance Assessment Form | Performance - Workplace-Based | PT | 2 | 0 | 0 | 0 | 0 | Joseph C (2011)^56^ |
| Clinical Performance | Clinical Performance Instrument (CPI) | Performance - Workplace-Based | PT | 0 | 0 | 0 | 1 | 0 | Kosmahl EM (2005)^57^ |
| Clinical Performance | Clinical Performance Instrument (CPI) | Performance - Workplace-Based | PT | 0 | 0 | 0 | 2 | 0 | Meiners KM (2017)^58^ |
| Clinical Performance | Clinical Performance Instrument (CPI) | Performance - Workplace-Based | PT | 0 | 2 | 0 | 0 | 0 | Sliwinski MM (2004)^59^ |
| Clinical Performance | Clinical Performance Instrument (CPI) | Performance - Workplace-Based | PT | 0 | 0 | 2 | 0 | 0 | Straube D (2003)^60^ |
| Clinical Performance | Clinical Performance Instrument (CPI) | Performance - Workplace-Based | PT | 0 | 0 | 0 | 1 | 0 | Thieman TJ (2003)^61^ |
| Clinical Performance | Clinical Performance Instrument (CPI) | Performance - Workplace-Based | PT | 0 | 2 | 0 | 0 | 0 | Vendrely A (2004)^62^ |
| Clinical performance | Clinical Performance Instrument (CPI) | Performance - OSCE/Simulation | PT | 0 | 2 | 0 | 0 | 0 | Wetherbee E (2018)^63^ |
| Clinical Performance | Clinical Performance Instrument (CPI) - PT | Performance - Workplace-Based | PT | 0 | 0 | 3 | 2 | 0 | Adams CL (2008)^64^ |
| Clinical Performance | Clinical Performance Instrument (CPI) - PT | Performance - Workplace-Based | PT | 0 | 0 | 0 | 2 | 0 | Bayliss J (2017)^65^ |
| Clinical Performance | Clinical Performance Instrument (CPI) – PT | Performance - Workplace-Based | PT | 0 | 2 | 0 | 0 | 0 | Norman K (2015)^66^ |
| Clinical Performance | Clinical Performance Instrument (CPI) (PT-CPI) | Performance - Workplace-Based | PT | 0 | 1 | 1 | 0 | 0 | Proctor PL (2010)^67^ |
| Clinical Performance | Clinical Performance Instrument (CPI) 3.0 | Performance - Workplace-Based | PT | 0 | 0 | 3 | 1 | 0 | Campbell DF (2025)^68^ |
| Clinical Performance | Clinical Performance Instrument (CPI) PT-CPI, Version 2006 | Performance - Workplace-Based | PT | 2 | 0 | 3 | 2 | 0 | Roach KE (2012)^69^ |
| Clinical Performance | Clinical Performance Instrument (version: PT CPI: Web) | Performance - Workplace-Based | PT | 0 | 0 | 2 | 0 | 0 | Wolden M (2021)^70^ |
| Clinical Performance | Clinical Performance Instruments (CPI) - PT and PTA | Performance - Workplace-Based | PT; PTA | 3 | 2 | 3 | 3 | 0 | Task Force (2002)^71^ |
| Clinical Performance | Common Assessment Form (CAF) | Performance - Workplace-Based | PT | 2 | 2 | 0 | 0 | 0 | Alpine LM (2021)^72^ |
| Clinical Performance | Common Assessment Form (CAF) | Performance - Workplace-Based | PT | 2 | 0 | 1 | 2 | 0 | Coote S (2007)^73^ |
| Clinical Performance | Common Evaluation Instrument | Performance - Workplace-Based | PT | 2 | 0 | 0 | 0 | 0 | Dickinson R (1973)^74^ |
| Clinical Performance | Evaluation Instrument for Clinical Education | Performance - Workplace-Based | PT | 1 | 0 | 0 | 0 | 0 | Kern BP (1971)^75^ |
| Clinical performance | Integrated Clinical Education Tool | Performance - Workplace-Based | PT | 2 | 1 | 1 | 1 | 0 | Becker M (2024)^76^ |
| Clinical Performance | Mini-CEX WebApp | Performance - Workplace-Based | PT | 3 | 2 | 3 | 1 | 3 | Fuentes-Cimma J (2023)^77^ |
| Clinical Performance | N/A | Performance - Workplace-Based | PT | 0 | 0 | 0 | 0 | 1 | Greene R (2006)^78^ |
| Clinical Performance | N/A | Performance - OSCE/Simulation | PT | 2 | 2 | 2 | 1 | 0 | Ladyshewsky R (2000)^79^ |
| Clinical Performance | N/A | Performance - OSCE/Simulation | PT | 2 | 0 | 0 | 0 | 3 | Stevens K (2013)^80^ |
| Clinical Performance | Physical Therapist Manual for the Assessment of Clinical Skills (PT MACS) | Performance - Workplace-Based | PT | 0 | 0 | 1 | 2 | 0 | Luedtke-Hoffmann K (2012)^81^ |
| Clinical Performance | Physical Therapist Manual for the Assessment of Clinical Skills (PT MACS) | Performance - Workplace-Based | PT | 3 | 0 | 0 | 0 | 0 | Stickley LA (2005)^82^ |
| Clinical Performance | Proposed Short Form of the Medical College of Virginia evaluation form | Performance - Workplace-Based | PT | 2 | 0 | 0 | 0 | 0 | Mays MJ (1973)^83^ |
| Clinical Reasoning | Clinical Reasoning Assessment Tool (CRAT) | Oral; Performance - OSCE/Simulation; Performance - Workplace-Based | PT | 0 | 0 | 0 | 1 | 0 | McDevitt A (2019)^84^ |
| Clinical Reasoning | Clinical Reasoning Assessment Tool (CRAT) | Oral; Performance - OSCE/Simulation; Performance - Workplace-Based | PT | 0 | 2 | 0 | 0 | 1 | McDevitt AD (2022)^85^ |
| Clinical Reasoning | Clinical Reasoning Assessment Tool (CRAT) | Performance - OSCE/Simulation; Performance - Workplace-Based | PT | 0 | 1 | 2 | 0 | 0 | Wolden B (2024)^86^ |
| Clinical Reasoning | Clinical Reasoning Assessment Tool (CRAT) | Performance - OSCE/Simulation; Performance - Workplace-Based | PT | 3 | 0 | 0 | 0 | 0 | Furze J (2015)^87^ |
| Clinical Reasoning | Script Concordance Test | Written - Multiple Choice and Multiple Choice with Likert Scale | PT | 3 | 2 | 1 | 2 | 0 | Kojich L (2024)^88^ |
| Clinical Reasoning | Structured Oral Self-directed Learning Examination (SOSLE) | Oral | ≥1 | 0 | 2 | 1 | 2 | 0 | Chapman JA (1993)^89^ |
| Clinical Reasoning | Think Aloud Standardized Patient Examination (TASPE) | Oral; Performance - OSCE/Simulation | PT | 1 | 0 | 2 | 0 | 0 | Fu W (2015)^90^ |
| Clinical Reasoning | Treatment Planning Assessment (TPA) | Performance - Workplace-Based | PT | 0 | 0 | 2 | 0 | 0 | Lewis LK (2008)^91^ |
| Clinical Reasoning | Unnamed OSCE (same as Figueroa-Arce 2022) | Performance - OSCE/Simulation | PT | 2 | 0 | 0 | 1 | 1 | Figueroa-González P (2023)^92^ |
| Clinical Reasoning | Unnamed OSCE (same as Figueroa-González 2023) | Written - Essay; Written - Other; Performance - OSCE/Simulation | PT | 1 | 0 | 0 | 0 | 1 | Figueroa-Arce N (2022)^93^ |
| Clinical Reasoning (pediatric physical therapy) | N/A | Performance - OSCE/Simulation; Performance - Workplace-Based | PT | 2 | 0 | 1 | 0 | 0 | Schreiber J (2020)^94^ |
| Clinical Reasoning and Ethical Decision-Making | N/A | Written – Other (cognitive map and short answer) | PT | 2 | 0 | 1 | 1 | 0 | Jones M (2014)^95^ |
| Communication Regarding Professional, Medicolegal, and Ethical issues | Debate | Oral | PT | 0 | 0 | 0 | 0 | 1 | Weeks BK (2016)^96^ |
| Cultural Competence | Modified Cultural Competence Continuum | Written - Essay | PT | 1 | 0 | 1 | 0 | 0 | Wong CK (2007)^97^ |
| Electrophysical Agents | N/A | Performance - OSCE/Simulation | PT | 1 | 0 | 2 | 0 | 0 | Gomez Miranda LA (2024)^98^ |
| Evidence Based Practice | Modified Fresno Test | Written - Essay | PT | 0 | 0 | 3 | 0 | 1 | Miller AH (2013)^99^ |
| Evidence Based Practice | Modified Fresno Test | Written - Other | PT | 3 | 2 | 2 | 0 | 0 | Silva AM (2016)^100^ |
| Evidence Based Practice | Modified Fresno Test | Written - Other | PT | 3 | 0 | 3 | 1 | 0 | Tilson JK (2010)^101^ |
| Evidence Based Practice | Modified Fresno Test - Chinese Version | Written – Other (short answer) | PT | 3 | 0 | 1 | 1 | 0 | Chen LY (2025)^102^ |
| Exercise Prescription | N/A | Performance - OSCE/Simulation | PT | 0 | 1 | 0 | 1 | 1 | Chong DYK (2020)^103^ |
| Exercise Prescription | N/A | Written - Essay | PT | 1 | 0 | 0 | 0 | 1 | Heijne A (2012)^104^ |
| Information Literacy Skills | Searching assignment | Written - Essay; Written - Other | ≥1 | 1 | 0 | 0 | 0 | 0 | Boruff JT (2011)^105^ |
| Information Literacy Skills | Valid Assessment of Learning in Undergraduate Education (VALUE) | Written - Essay | PT | 1 | 0 | 1 | 0 | 0 | Turbow DJ (2016)^106^ |
| Interprofessional Collaborative Care Skills | Faculty Observation Assessment (FOA)/Standardized Patient Observation Assessment (SPOA) | Performance - OSCE/Simulation | IPE | 1 | 0 | 0 | 0 | 0 | Pabian PS (2022)^107^ |
| Interprofessional Collaborative Care Skills | N/A | Performance - OSCE/Simulation | IPE | 2 | 0 | 0 | 0 | 0 | Smeets HWH (2024)^108^ |
| Interprofessional Collaborative Care Skills | N/A | Performance - OSCE/Simulation | IPE | 2 | 2 | 0 | 0 | 0 | Smeets HWH (2025)^109^ |
| Interprofessional Collaborative Care Skills | Team Observation Instrument-Revised (TOI-R) | Performance - OSCE/Simulation | IPE | 0 | 0 | 0 | 1 | 0 | Lysaght C (2022)^110^ |
| Interprofessional Discharge Planning | Standardized Patient Team Experience (SPTE) Feedback and Reflection Form | Performance - OSCE/Simulation | IPE | 3 | 3 | 0 | 0 | 2 | East L (2025)^111^ |
| Manual Physical Therapy Skills | N/A | Performance - OSCE/Simulation | PT | 0 | 0 | 0 | 0 | 1 | Pérez-Guillén S (2022)^112^ |
| Medical Screening | N/A | Performance - OSCE/Simulation | PT | 0 | 1 | 0 | 0 | 0 | Johnston TE (2018)^113^ |
| Musculoskeletal Competency | Musculoskeletal OSCE | Performance - OSCE/Simulation | PT | 1 | 2 | 2 | 0 | 0 | Swift M (2013)^114^ |
| Musculoskeletal Competency | Musculoskeletal OSCE | Performance - OSCE/Simulation | PT | 0 | 0 | 2 | 0 | 2 | Swift M (2016)^115^ |
| Musculoskeletal Competency | N/A | Written - Essay; Performance - OSCE/Simulation | PT | 0 | 0 | 2 | 0 | 0 | Seymour CJ (1996)^116^ |
| Musculoskeletal Competency (examination, evaluation, and intervention) | Unnamed Musculoskeletal OSCE | Performance - OSCE/Simulation | PT | 0 | 0 | 2 | 2 | 0 | Wessel J (2003)^117^ |
| Neurologic Physical Therapy Skills | N/A | Performance - OSCE/Simulation | PT | 1 | 0 | 0 | 0 | 2 | Tunney N (2017)^118^ |
| Neurologic Physical Therapy Skills | Neuromuscular OSCE | Performance - OSCE/Simulation | PT | 3 | 1 | 3 | 1 | 0 | Gorman SL (2010)^119^ |
| Neurologic Physical Therapy Skills | Peer-simulation practical examination | Performance - OSCE/Simulation | PT | 0 | 2 | 0 | 2 | 0 | Ryall T (2025)^120^ |
| Neurologic Physical Therapy Skills (NDT, PNF) | N/A (same as Garcia-Ros 2021) | Performance - OSCE/Simulation | PT | 2 | 0 | 3 | 0 | 1 | Garcia-Ros R (2024)^121^ |
| Neurologic Physical Therapy Skills (PNF) | N/A | Performance - OSCE/Simulation | PT | 0 | 0 | 1 | 0 | 0 | Gittinger FP (2022)^122^ |
| Neurological Physical Therapy Skills (NDT, PNF) | N/A (same as Garcia-Ros 2024) | Performance - OSCE/Simulation | PT | 2 | 0 | 0 | 0 | 1 | Garcia-Ros R (2021)^123^ |
| Patient Education | Patient Education Performance Tool | Performance - OSCE/Simulation | PT | 3 | 0 | 2 | 0 | 0 | Forbes R (2019)^124^ |
| Patient Interview Skills | ECHOWS tool | Performance - OSCE/Simulation | PT | 0 | 1 | 2 | 1 | 0 | Boissonnault JS (2016)^125^ |
| Patient Interview Skills | ECHOWS Tool | Performance - Workplace-Based | PT | 0 | 0 | 1 | 0 | 0 | Seif GA (2019)^126^ |
| Pelvic Floor Muscle Examination | N/A | Performance - OSCE/Simulation | PT | 1 | 0 | 0 | 0 | 0 | Fisher KA (2008)^127^ |
| Practice Development | Term-paper assignments | Written - Essay | ≥1 | 0 | 0 | 1 | 0 | 0 | Williams R (1991)^128^ |
| Procedural Skills | Assessment of Procedural Skills in Physical Therapy Education (APSPT) | Performance - OSCE/Simulation | PT | 0 | 0 | 3 | 0 | 0 | Sattelmayer KM (2020)^129^ |
| Professional behavior | Academic Professional Behavior Assessment Tool | Performance - Classroom-Based | PT | 2 | 0 | 0 | 2 | 1 | Dorsey LL (2018)^130^ |
| Professionalism | RISE-CI Assessment Tool (RISE = Respect for Others, Integrity & Compliance, Self-Awareness & Commitment to Development, and Engagement & Work Ethic. CI = Clinical Instructor) | Performance - Workplace-Based | PT | 2 | 2 | 0 | 0 | 0 | Edgeworth Ditwiler R  (2023)^131^ |
| Professionalism | Standards of Professionalism rubric | Other: Performance - Longitudinal Classroom Behaviors | PT | 2 | 2 | 0 | 2 | 0 | Nesbit KC (2021)^132^ |
| Reflection | Reflective Journal | Written - Essay | PT | 2 | 0 | 2 | 0 | 0 | Plack MM (2005)^133^ |
| Spanish Language Proficiency | Physical Therapy Spanish Proficiency Measure (PT-SPM) | Performance - Workplace-Based | PT | 2 | 0 | 0 | 0 | 0 | Pechak C (2019)^134^ |
| Sterile-Technique Hand Washing | N/A | Performance - OSCE/Simulation | PT | 2 | 2 | 2 | 0 | 0 | Riolo L (1997)^135^ |
| Teamwork Skills | N/A | Performance - OSCE/Simulation | IPE | 1 | 0 | 2 | 1 | 0 | Emmert MC (2015)^136^ |
| Teamwork Skills | Team Performance Observation Tool (with scenario-specific targeted behavioral markers) | Performance - OSCE/Simulation | IPE | 1 | 0 | 2 | 2 | 0 | Zhang C (2015)^137^ |
| Vestibular Rehabilitation Skills | N/A | Performance - OSCE/Simulation | PT | 3 | 0 | 0 | 0 | 0 | Tappan RS (2020)^138^ |
| X-Ray Interpretation | N/A | Written - Other | PT | 1 | 0 | 0 | 0 | 1 | Clark CR (2016)^139^ |

References:

1. Kinirons SA, Reddin VM. Early identification of students at risk for academic difficulty in a Doctor of Physical Therapy program. *J Allied Health*. 2023;52(1):e9-e15.

2. Zhang G, Fenderson BA, Schmidt RR, Veloski JJ. Equivalence of students’ scores on timed and untimed anatomy practical examinations. *Anat Sci Educ*. 2013;6(5):281-285. doi:10.1002/ase.1357

3. Fabrizio PA. Oral anatomy laboratory examinations in a physical therapy program. *Anat Sci Educ*. 2013;6(4):271-276. doi:10.1002/ase.1339

4. Daly FJ. Use of electronic anatomy practical examinations for remediating “at risk” students. *Anat Sci Educ*. 2010;3(1):46-49. doi:10.1002/ase.120

5. Roth HR, Holland EE, Goh L, Wong E, McGaghie WC, Tappan RS. Systematic development and validity evidence for a checklist to assess bed mobility skills among physical therapy students. *J Allied Health*. 2024;53(2):122-129.

6. Pata R, O’Sullivan S, Peterson D, Superchi L, Feinn R. Simulation-Based Cardiopulmonary Examination Skills Checklist: Assessment of inter-rater reliability. *Cardiopulm Phys Ther J Lippincott Williams Wilkins*. 2024;35(3):113-123. doi:10.1097/CPT.0000000000000251

7. Balaraman T, Śliwmski Z. The effectiveness of a case based online video assignment in improving practical performance of physiotherapy students - a quasi experimental study. *Fizjoterapia Pol*. 2021;21(2):30-34.

8. Kulkarni M, Sinha R, Sinha S, Mahajan A. Perception of undergraduate physiotherapy students toward objectively structured practical examination checklist as a tool for assessment and learning active cycle of breathing techniques skill: A cross-sectional descriptive study. *Indian J Phys Ther Res*. 2023;5(2):182-186. doi:10.4103/ijptr.ijptr_108_23

9. Silva CCBM, Lunardi AC, Mendes FAR, Souza FFP, Carvalho CRF. Objective structured clinical evaluation as an assessment method for undergraduate chest physical therapy students: A cross-sectional study. *Braz J Phys Ther*. 2011;15(6):481-486. doi:10.1590/S1413-35552011005000033

10. Dalton M, Davidson M, Keating J. The Assessment of Physiotherapy Practice (APP) is a valid measure of professional competence of physiotherapy students: A cross-sectional study with Rasch analysis. *J Physiother Elsevier*. 2011;57(4):239-246.

11. Dalton M, Davidson M, Keating JL. The Assessment of Physiotherapy Practice (APP) is a reliable measure of professional competence of physiotherapy students: A reliability study. *J Physiother Elsevier*. 2012;58(1):49-56. doi:10.1016/S1836-9553(12)70072-3

12. Fulton T, Myatt K, Kirwan GW, Clark CR, Dalton M. Rating of physiotherapy student clinical performance in a paediatric setting: are assessors consistent in their rating of a simulated clinical student performance? *BMC Med Educ*. 2023;23(1):280. doi:10.1186/s12909-023-04149-9

13. Jones A, Mandrusiak A, Judd B, Gordon C, Alison J. Investigating a physiotherapy clinical simulation assessment tool using the Delphi approach. *Internet J Allied Health Sci Pract*. 2017;15(3):1-6.

14. Judd BK, Scanlan JN, Alison JA, Waters D, Gordon CJ. The validity of a professional competence tool for physiotherapy students in simulation-based clinical education: a Rasch analysis. *BMC Med Educ*. 2016;16:196. doi:10.1186/s12909-016-0718-x

15. Kirwan GW, Clark CR, Dalton M. Rating of physiotherapy student clinical performance: Is it possible to gain assessor consistency? *BMC Med Educ*. 2019;19(1):32. doi:10.1186/s12909-019-1459-4

16. Lo K, Osadnik C, Leonard M, Maloney S. Differences in student and clinician perceptions of clinical competency in undergraduate physiotherapy. *N Z J Physiother*. 2015;43(1):11-15. doi:10.15619/NZJP/43.1.02

17. Murphy S, Dalton M, Dawes D. Assessing physical therapy students’ performance during clinical practice. *Physiother Can*. 2014;66(2):169-176. doi:10.3138/ptc.2013-26

18. Reubenson A, Ng L, Gucciardi DF. The Assessment of Physiotherapy Practice tool provides informative assessments of clinical and professional dimensions of student performance in undergraduate placements: A longitudinal validity and reliability study. *J Physiother*. 2020;66(2):113-119. doi:10.1016/j.jphys.2020.03.009

19. Hu J, Jones AYM, Zhou X, et al. Acceptance of the “Assessment of Physiotherapy Practice (Chinese)” as a standardised evaluation of professional competency in Chinese physiotherapy students: an observational study. *BMC Med Educ*. 2020;20(1):108. doi:10.1186/s12909-020-02026-3

20. Schwartz D, Jacob T. Establishing the reliability of a tool for assessing Israeli physical therapy students’ clinical performance. *J Phys Ther Educ Lippincott Williams Wilkins*. 2019;33(3):243-248. doi:10.1097/JTE.0000000000000093

21. Çelik Hİ, Ozturk D, Sari M, Bulut N, Bek N. Psychometric and edumetric properties of the Turkish version of the assessment of physiotherapy practice. *BMC Med Educ*. 2024;24(1):1162. doi:10.1186/s12909-024-06180-w

22. Muhamad Z, Ramli A, Amat S. Validity and reliability of the Clinical Competency Evaluation Instrument for use among physiotherapy students: Pilot study. *Sultan Qaboos Univ Med J*. 2015;15(2):e266-74.

23. Morris J. Audit of use of a common undergraduate physiotherapy clinical assessment form. *Int J Ther Rehabil*. 2006;13(9):407-413. doi:10.12968/ijtr.2006.13.9.21785

24. Loomis J. Evaluating clinical competence of physical therapy students: The development of an instrument... part 1. *Physiother Can*. 1985;37(2):83-89.

25. Loomis J. Evaluating clinical competence of physical therapy students: Assessing the reliability, validity and usability of a new instrument... part 2. *Physiother Can*. 1985;37(2):91-98.

26. Cross V, Hicks C, Barwell F. Exploring the gap between evidence and judgement: Using video vignettes for practice-based assessment of physiotherapy undergraduates. *Assess Eval High Educ*. 2001;26(3):189-212.

27. Panzarella KJ, Manyon AT. A model for integrated assessment of clinical competence. *J Allied Health*. 2007;36(3):157-164.

28. Panzarella KJ, Manyon AT. Using the Integrated Standardized Patient Examination to assess clinical competence in physical therapist students. *J Phys Ther Educ*. 2008;22(3):24-32. doi:10.1097/00001416-200810000-00004

29. Torres-Narváez MR, Vargas-Pinilla OC, Rodríguez-Grande EI. Validity and reproducibility of a tool for assessing clinical competencies in physical therapy students. *BMC Med Educ*. 2018;18(1):280. doi:10.1186/s12909-018-1377-x

30. Costello E, Plack M, Maring J. Validating a standardized patient assessment tool using published professional standards. *J Phys Ther Educ*. 2011;25(3):30-46. doi:10.1097/00001416-201107000-00007

31. Kanada Y. Standardizing the assessment of the clinical abilities of physical therapists and occupational therapists using OSCE. *J Phys Ther Sci*. Published online 2012.

32. Kanada Y, Sakurai H, Sugiura Y. Difficulty levels of OSCE items related to examination and measurement skills. *J Phys Ther Sci*. 2015;27(3):715-718. doi:10.1589/jpts.27.715

33. Oldmeadow L. Developing clinical competence: A mastery pathway. *Aust J Physiother*. 1996;42(1):37-44. doi:10.1016/s0004-9514(14)60439-6

34. Rheault W, Coulson E. Use of the Rasch model in the development of a clinical competence scale. *J Phys Ther Educ Am Phys Ther Assoc Educ Sect*. 1991;5(1):10-13.

35. Sakurai H, Kanada Y, Sugiura Y, et al. Standardization of clinical competency evaluation in the education of physical therapists and occupational therapists - Establishment of an OSCE compliant education system -. *J Phys Ther Sci*. 2013;25(1):101-107. doi:10.1589/jpts.25.101

36. Flew B, Chipchase L, Lee D, McClelland JA. Feasibility of an online clinical assessment of competence in physiotherapy students. *Physiother Theory Pr*. 2025;41(3):508-521. doi:10.1080/09593985.2024.2344024

37. Martiáñez-Ramírez NL, Pineda-Galán C, Rodríguez-Bailón M, Romero-Galisteo RP. Competence assessment rubric in the Physiotherapy Practicum. *PLoS One*. 2022;17(2):e0264120. doi:10.1371/journal.pone.0264120

38. Panzarella KJ. Beginning with the end in mind: Evaluating outcomes of cultural competence instruction in a Doctor of Physical Therapy programme. *Disabil Rehabil*. 2009;31(14):1144-1152. doi:10.1080/09638280902773745

39. White LW, Jordan KE, McDermott H. Assessment of student readiness for clinical education in mixed-mode curriculum delivery: A case study. *Qual Assur Educ Int Perspect*. 2023;31(1):151-166. doi:10.1108/QAE-02-2022-0044

40. Ladyshewsky R, Baker R, Jones M, Nelson L. Reliability and validity of an extended simulated patient case: a tool for evaluation and research in physiotherapy. *Physiother Theory Pract*. 2000;16(1):15-25. doi:10.1080/095939800307575

41. Hayward LM, Blackmer B, Markowski A. Standardized patients and communities of practice: a realistic strategy for integrating the core values in a physical therapist education program. *J Phys Ther Educ Am Phys Ther Assoc Educ Sect*. 2006;20(2):29-37. doi:10.1097/00001416-200607000-00005

42. Hayward LM, Blackmer B. A model for teaching and assessing core values development in Doctor of Physical Therapy students. *J Phys Ther Educ*. 2010;24(3):16-26. doi:10.1097/00001416-201007000-00003

43. O’Malley E, Scanlon AM, Alpine L, McMahon S. Enabling the feedback process in work-based learning: An evaluation of the 5 Minute Feedback Form. *Assess Eval High Educ*. 2021;46(7):1020-1034. doi:10.1080/02602938.2020.1842852

44. Hrachovy J, Clopton N, Baggett K, Garber T, Cantwell J, Schreiber J. Use of the Blue MACS: Acceptance by clinical instructors and self-reports of adherence. *Phys Ther*. 2000;80(7):652-661.

45. Mori B, Norman KE, Brooks D, Herold J, Beaton DE. Canadian Physiotherapy Assessment of Clinical Performance: Face and content validity. *Physiother Can*. 2016;68(1):64-72. doi:10.3138/ptc.2015-35E

46. Mori B, Norman KE, Brooks D, Herold J, Beaton DE. Evidence of reliability, validity, and practicality for the Canadian Physiotherapy Assessment of Clinical Performance. *Physiother Can*. 2016;68(2):156-169. doi:10.3138/ptc.2014-43E

47. Yeldon J, Wilson R, Laferrière J, et al. Let’s talk about the talk: Exploring the experience of discussing student performance at the mid- and final points of the clinical internship. *Physiother Can*. 2018;70(3):240-248. doi:10.3138/ptc.2016-96

48. Mori B, Brooks D, Norman KE, Herold J, Beaton DE. Development of the Canadian Physiotherapy Assessment of Clinical Performance: A new tool to assess physiotherapy students’ performance in clinical education. *Physiother Can*. 2015;67(3):281-289. doi:10.3138/ptc.2014-29E

49. Mori B, Daly A, Norman KE, Wojkowski S. The development of the Canadian Physiotherapy Assessment of Clinical Performance (ACP) 2.0 - Alignment with the 2017 Competency Profile. *Physiother Can*. 2024;76(1):111-120. doi:10.3138/ptc-2021-0077

50. Naylor S, Norris M, Williams A. Does ethnicity, gender or age of physiotherapy students affect performance in the final clinical placements? An exploratory study. *Physiotherapy*. 2014;100(1):9-13. doi:10.1016/j.physio.2013.05.004

51. Meldrum D, Lydon A, Loughnane M, et al. Assessment of undergraduate physiotherapist clinical performance: Investigation of educator inter-rater reliability. *Physiotherapy*. 2008;94(3):212-219. doi:10.1016/j.physio.2008.03.003

52. Birkmeier M, Wheeler E, McGregor Garske H, et al. Feasibility of use of the Clinical Internship Evaluation Tool in full-time clinical education experiences: A multi-institutional study. *J Phys Ther Educ*. 2022;36(3):263-271. doi:10.1097/JTE.0000000000000237

53. Fitzgerald LM, Delitto A, Irrgang JJ. Validation of the Clinical Internship Evaluation Tool. *Phys Ther*. 2007;87(7):844-860. doi:10.2522/ptj.20060054

54. North S, Sharp A. Embracing change in the pursuit of excellence: Transitioning to the Clinical Internship Evaluation Tool for student clinical performance assessment. *J Phys Ther Educ*. 2020;34(4):313-320. doi:10.1097/JTE.0000000000000154

55. Joseph C, Frantz J, Hendricks C, Smith M. Evaluation of a new clinical performance assessment tool: A reliability study. *South Afr J Physiother*. 2012;68(3):15-19.

56. Joseph C, Hendricks C, Frantz J. Exploring the key performance areas and assessment criteria for the evaluation of students’ clinical performance: A Delphi study. *South Afr J Physiother*. 2011;67(2):9-15.

57. Kosmahl EM. Factors related to physical therapist license examination scores. *J Phys Ther Educ Am Phys Ther Assoc Educ Sect*. 2005;19(2):52-56. doi:10.1097/00001416-200507000-00007

58. Meiners KM, Rush DK. Clinical performance and admission variables as predictors of passage of the National Physical Therapy Examination. *J Allied Health*. 2017;46(3):164-170.

59. Sliwinski MM, Schultze K, Hansen RL, Malta S, Babyar SR. Clinical performance expectations: A preliminary study comparing physical therapist students, clinical instructors, and academic faculty. *J Phys Ther Educ Am Phys Ther Assoc Educ Sect*. 2004;18(1):50-57. doi:10.1097/00001416-200401000-00007

60. Straube D, Campbell SK. Rater discrimination using the visual analog scale of the Physical Therapist Clinical Performance Instrument. *J Phys Ther Educ Am Phys Ther Assoc Educ Sect*. 2003;17(1):33-38.

61. Thieman TJ, Weddle ML, Moore MA. Predicting academic, clinical, and licensure examination performance in a professional (entry-level) master’s degree program in physical therapy. *J Phys Ther Educ Am Phys Ther Assoc Educ Sect*. 2003;17(2):32-37.

62. Vendrely A, Carter R. The influence of training on the rating of physical therapist student performance in the clinical setting. *J Allied Health*. 2004;33(1):62-69.

63. Wetherbee E, Dupre AM, Feinn RS, Roush S. Relationship between narrative comments and ratings for entry-level performance on the Clinical Performance Instrument: A call to rethink the Clinical Performance Instrument. *J Phys Ther Educ Lippincott Williams Wilkins*. 2018;32(4):333-343. doi:10.1097/JTE.0000000000000060

64. Adams CL, Glavin K, Hutchins K, Lee T, Zimmerman C. An evaluation of the internal reliability, construct validity, and predictive validity of the Physical Therapist Clinical Performance Instrument (PT CPI). *J Phys Ther Educ Am Phys Ther Assoc Educ Sect*. 2008;22(2):42-50. doi:10.1097/00001416-200807000-00007

65. Bayliss J, Thomas RM, Eifert-Mangine M. Pilot Study: What measures predict first time pass rate on the National Physical Therapy Examination? *Internet J Allied Health Sci Pract*. 2017;15(4):2-12.

66. Norman KE, Booth R. Observations and performances “with distinction” by physical therapy students in clinical education: Analysis of checkboxes on the Physical Therapist Clinical Performance Instrument (PT-CPI) over a 4-Year period. *Physiother Can*. 2015;67(1):17-29. doi:10.3138/ptc.2013-64

67. Proctor PL, Dal Bello-Haas VP, McQuarrie AM, Sheppard MS, Scudds RJ. Scoring of the physical therapist clinical performance instrument (PT-CPI): Analysis of 7 years of use. *Physiother Can*. 2010;62(2):147-154. doi:10.3138/physio.62.2.147

68. Campbell DF, Alameri M, Macahilig-Rice F, Witkin SE, Hellman NG. Validation of the Revised American Physical Therapy Association Physical Therapist Clinical Performance Instrument 3.0. *Phys Ther*. Published online 2025. doi:10.1093/ptj/pzaf015

69. Roach KE, Frost JS, Francis NJ, Giles S, Nordrum JT, Delitto A. Validation of the Revised Physical Therapist Clinical Performance Instrument (PT CPI): Version 2006. *Phys Ther*. 2012;92(3):416-428. doi:10.2522/ptj.20110129

70. Wolden M, Drevyn E, Flom-Meland C, Gusman LN. Evaluation and modification of the Physical Therapist Clinical Performance Instrument. *J Phys Ther Educ Lippincott Williams Wilkins*. 2021;35(2):85-94. doi:10.1097/JTE.0000000000000180

71. The development and testing of APTA clinical performance instruments. *Phys Ther*. 2002;82(4):329-353.

72. Alpine LM, O’Connor A, McGuinness M, Barrett EM. Performance-based assessment during clinical placement: Cross-sectional investigation of a training workshop for practice educators. *Nurs Health Sci*. 2021;23(1):113-122. doi:10.1111/nhs.12768

73. Coote S, Alpine L, Cassidy C, et al. The development and evaluation of a Common Assessment Form for physiotherapy practice education in Ireland. *Physiother Irel*. 2007;28(2):6-10.

74. Dickinson R, DiMarino J, Pfitzenmaier J. A common evaluation instrument. *Phys Ther*. 1973;53(10):1075-1080. doi:10.1093/ptj/53.10.1075

75. Kern BP, Mickelson JM. The development and use of an evaluation instrument for clinical education. *Phys Ther*. 1971;51(5):540-546. doi:10.1093/ptj/51.5.540

76. Becker M, Shields RK, Sass KJ. Psychometric analysis of an integrated clinical education tool for physical therapists. *J Phys Ther Educ*. 2024;38(4):277-284. doi:10.1097/JTE.0000000000000341

77. Fuentes-Cimma J, Fuentes-López E, Isbej Espósito L, et al. Utility analysis of an adapted Mini-CEX WebApp for clinical practice assessment in physiotherapy undergraduate students. *Front Educ*. 2023;8. doi:10.3389/feduc.2023.943709

78. Greene R, Rogers GL. Justifying core faculty assessment of students’ clinical performance using cognitive flexibility theory: a case example. *Internet J Allied Health Sci Pract*. 2006;4(3):5p-5p.

79. Ladyshewsky R, Baker R, Jones M, Nelson L. Evaluating clinical performance in physical therapy with simulated patients. *J Phys Ther Educ Am Phys Ther Assoc Educ Sect*. 2000;14(1):31-37.

80. Stevens K, Henderson H, Hawthorne K, Carlson J. A comparison of methods for setting passing scores in standardized simulated patient experiences in physical therapist education. *J Phys Ther Educ*. 2013;27(3):78-81.

81. Luedtke-Hoffmann K, Dillon L, Utsey C, Tomaka J. Is there a relationship between performance during physical therapist clinical education and scores on the National Physical Therapy Examination (NPTE)? *J Phys Ther Educ Am Phys Ther Assoc Educ Sect*. 2012;26(2):41-49.

82. Stickley LA. Content validity of a clinical education performance tool: The Physical Therapist Manual for the Assessment of Clinical Skills. *J Allied Health*. 2005;34(1):24-30.

83. Mays MJ. Reliability of a method of evaluating the clinical performance of a physical therapy student. *Phys Ther*. 1973;53(12):1298-1306.

84. McDevitt A, Rapport MJ, Jensen G, Furze J. Utilization of the Clinical Reasoning Assessment Tool across a physical therapy curriculum: Application for teaching, learning, and assessment. *J Phys Ther Educ Lippincott Williams Wilkins*. 2019;33(4):335-342. doi:10.1097/JTE.0000000000000110

85. McDevitt AD, Rapport MJD, Rodriguez JD, Miller M. Faculty perceptions on use of the Clinical Reasoning Assessment Tool to support learning in physical therapist students: A qualitative study. *J Phys Ther Educ Lippincott Williams Wilkins*. 2022;36(1):57-64. doi:10.1097/JTE.0000000000000207

86. Wolden B, Wolden M, Furze J, McDevitt A. Advancing consistency in education: A reliability analysis of the Clinical Reasoning Assessment Tool. *J Phys Ther Educ*. Published online 2024. doi:10.1097/JTE.0000000000000365

87. Furze J, Gale JR, Black L, Cochran TM, Jensen GM. Clinical reasoning: Development of a grading rubric for student assessment. *J Phys Ther Educ*. 2015;29(3):34-45. doi:10.1097/00001416-201529030-00006

88. Kojich L, Miller SA, Axman K, Eacret T, Koontz JA, Smith C. Evaluating clinical reasoning in first year DPT students using a script concordance test. *BMC Med Educ*. 2024;24(1):329. doi:10.1186/s12909-024-05281-w

89. Chapman JA, Westmorland MG, Norman GR, Durrell K, Hall A. The structured oral self-directed learning evaluation: One method of evaluating the clinical reasoning skills of occupational therapy and physiotherapy students. *Med Teach*. 1993;15(2-3):223-236. doi:10.3109/01421599309006717

90. Fu W. Development of an innovative tool to assess student physical therapists’ clinical reasoning competency. *J Phys Ther Educ*. 2015;29(4):14-26. doi:10.1097/00001416-201529040-00004

91. Lewis LK, Stiller K, Hardy F. A clinical assessment tool used for physiotherapy students--is it reliable? *Physiother Theory Pr*. 2008;24(2):121-134. doi:10.1080/09593980701508894

92. Figueroa-González P, Figueroa-Arce N, Gómez-Miranda L, Gutiérrez-Arias R, Contreras-Pizarro V. Satisfaction level and correlation between performance and self-evaluation of physical therapy students in an objective structured clinical examination (OSCE) designed to assess clinical reasoning. *Rev Fac Med*. 2023;71(4). doi:10.15446/revfacmed.v71n4.107397

93. Figueroa-Arce N, Figueroa-González P, Gómez-Miranda L, Gutiérrez-Arias R, Contreras-Pizarro V. Implementation of an Objective structured clinical examination (OSCE) as a tool to evaluate the development of clinical reasoning in physical therapy students. *Rev Fac Med*. 2022;70(2). doi:10.15446/revfacmed.v70n2.90746

94. Schreiber J, Gagnon K, Kendall E, LaForme Fiss A, Rapport MJ, Wynarczuk KD. Development of a grading rubric to assess learning in pediatric physical therapy education. *Pediatr Phys Ther*. 2020;32(1):70-79. doi:10.1097/PEP.0000000000000667

95. Jones M, van Kessel G, Swisher L, Beckstead J, Edwards I. Cognitive maps and the structure of observed learning outcome assessment of physiotherapy students’ ethical reasoning knowledge. *Assess Eval High Educ*. 2014;39(1):1-20. doi:10.1080/02602938.2013.772951

96. Weeks BK, Laakso L. Using debates as assessment in a physiotherapy capstone course: A case example. *J Univ Teach Learn Pract*. 2016;13(3). https://search.ebscohost.com/login.aspx?direct=true&db=eric&AN=EJ1110546&site=ehost-live

97. Wong CK, Blissett S. Assessing performance in the area of cultural competence: an analysis of reflective writing. *J Phys Ther Educ Am Phys Ther Assoc Educ Sect*. 2007;21(1):40-47. doi:10.1097/00001416-200701000-00006

98. Gomez Miranda LA, Amigo Reyes T, de la Barra Ortiz HA. Validating an objective structured clinical examination to enhance assessment of clinical skills in physical therapy students. *J Adv Pharm Educ Res*. 2024;14(2):16-26. doi:10.51847/c2DlK9b9pQ

99. Miller AH, Cummings N, Tomlinson J. Measurement error and detectable change for the modified Fresno Test in first-year entry-level physical therapy students. *J Allied Health*. 2013;42(3):169-174.

100. Silva AM, Costa LCM, Comper ML, Padula RS. Cross-cultural adaptation and reproducibility of the Brazilian-Portuguese version of the modified FRESNO Test to evaluate the competence in evidence based practice by physical therapists. *Braz J Phys Ther*. 2016;20(1):26-47. doi:10.1590/bjpt-rbf.2014.0140

101. Tilson JK. Validation of the modified Fresno Test: Assessing physical therapists’ evidence based practice knowledge and skills. *BMC Med Educ*. 2010;10(1). doi:10.1186/1472-6920-10-38

102. Chen LY, Lue YJ, Wu PH, Kuo YL. Cross-cultural adaptation and validation of the Chinese version of the modified Fresno test for physical therapists. *BMC Med Educ*. 2025;25(1):1. doi:10.1186/s12909-024-06615-4

103. Chong DYK, Tam B, Yau SY, Wong AYL. Learning to prescribe and instruct exercise in physiotherapy education through authentic continuous assessment and rubrics. *BMC Med Educ*. 2020;20(1):258. doi:10.1186/s12909-020-02163-9

104. Heijne A, Nordgren B, Hagströmer M, Fridén C. Assessment by portfolio in a physiotherapy programme. *Adv Physiother*. 2012;14(1):38-46. doi:10.3109/14038196.2012.661458

105. Boruff JT, Thomas A. Integrating evidence-based practice and information literacy skills in teaching physical and occupational therapy students. *Health Inf Libr J*. 2011;28(4):264-272. doi:10.1111/j.1471-1842.2011.00953.x

106. Turbow DJ, Evener J. Norming a VALUE rubric to assess graduate information literacy skills. *J Med Libr Assoc*. 2016;104(3):209-214. doi:10.3163/1536-5050.104.3.005

107. Pabian PS, Kay D, Neely L, Whitworth J. An interprofessional education approach to pain management through a standardized patient encounter. *J Interprofessional Educ Pract*. 2022;29. doi:10.1016/j.xjep.2022.100568

108. Smeets HWH, Delnoij LEC, Sluijsmans DMA, Moser A, van Merriënboer JJG. From individual to interprofessional: characteristics of assessment tasks to assess interprofessional collaboration in healthcare education. *J Interprof Care*. 2024;38(5):907-917. doi:10.1080/13561820.2024.2381058

109. Smeets HWH, Delnoij LEC, Sluijsmans DMA, Moser A, van Merrienboer JJG. *The Balancing Act of Assessment Validity in Interprofessional Healthcare Education: A Qualitative Evaluation Study.* Taylor & Francis Ltd; 2025:99-112. https://turing.library.northwestern.edu/login?url=https://search.ebscohost.com/login.aspx?direct=true&db=rzh&AN=181729679&site=ehost-live

110. Lysaght C, Lin CC, Stokes CK, et al. Interprofessional collaborative care skills and behaviors: Perception differences between allied health students and an independent observer. *J Allied Health*. 2022;51(4):e77-e84.

111. East L, Stevens A, Hageman H, et al. Validity evidence for a feedback tool for an interprofessional standardized patient experience: A qualitative pilot study with evaluation of content, response processes, consequences of testing, and feedback quality. *J Interprofessional Educ Pract*. 2025;40. doi:10.1016/j.xjep.2025.100749

112. Pérez-Guillén S, Carrasco-Uribarren A, Celis CL, González-Rueda V, Rodríguez-Rubio PR, Cabanillas-Barea S. Students’ perceptions, engagement and satisfaction with the use of an e-rubric for the assessment of manual skills in physiotherapy. *BMC Med Educ*. 2022;22(1):623. doi:10.1186/s12909-022-03651-w

113. Johnston TE. Assessment of medical screening and clinical reasoning skills by physical therapy students in a simulated patient encounter. *Internet J Allied Health Sci Pract*. 2018;16(2):1-9.

114. Swift M, Spake E, Gajewski BJ. The reliability of a Musculoskeletal Objective Structured Clinical Examination in a professional physical therapist program. *J Phys Ther Educ*. 2013;27(2):41-48. doi:10.1097/00001416-201301000-00011

115. Swift M, Spake E, Kohia M. Examiner fatigue and ability to concentrate in objective structured clinical examinations for physical therapist students. *J Allied Health*. 2016;45(1):62-70.

116. Seymour CJ, Dybel GJ. Developing skillful clinical decision making: Evaluation of two classroom teaching strategies. *J Phys Ther Educ Am Phys Ther Assoc Educ Sect*. 1996;10(2):77-81.

117. Wessel J, Williams R, Finch E, Gémus M. Reliability and validity of an objective structured clinical examination for physical therapy students. *J Allied Health*. 2003;32(4):266-269.

118. Tunney N, Perlow E. Student and examiner perceptions of an innovative model for assessment of neuromuscular clinical competence in a professional physical therapist education program. *J Phys Ther Educ Am Phys Ther Assoc Educ Sect*. 2017;31(3):91-99. doi:10.1097/00001416-201731030-00016

119. Gorman SL, Lazaro R, Fairchild J, Kennedy B. Development and implementation of an Objective Structured Clinical Examination (OSCE) in neuromuscular physical therapy. *J Phys Ther Educ*. 2010;24(3):62-68. doi:10.1097/00001416-201007000-00008

120. Ryall T, Preston E, Bissett B. Can classroom-based peer patient simulation predict real-life clinical performance in physiotherapy students? *Heliyon*. 2025;11(6). doi:10.1016/j.heliyon.2025.e43027

121. Garcia-Ros R, Ruescas-Nicolau MA, Cezón-Serrano N, Flor-Rufino C, Martin-Valenzuela CS, Sánchez-Sánchez ML. Improving assessment of procedural skills in health sciences education: a validation study of a rubrics system in neurophysiotherapy. *BMC Psychol*. 2024;12(1):147. doi:10.1186/s40359-024-01643-7

122. Gittinger FP, Lemos M, Neumann JL, et al. Interrater reliability in the assessment of physiotherapy students. *BMC Med Educ*. 2022;22(1):186. doi:10.1186/s12909-022-03231-y

123. García-Ros R, Ruescas-Nicolau MA, Cezón-Serrano N, et al. Students’ perceptions of instructional rubrics in neurological Pphysical therapy and their effects on students’ engagement and course satisfaction. *Int J Env Res Public Health*. 2021;18(9). doi:10.3390/ijerph18094957

124. Forbes R, Mandrusiak A. Development and reliability testing of a patient education performance tool for physical therapy students. *J Phys Ther Educ Lippincott Williams Wilkins*. 2019;33(1):64-69. doi:10.1097/JTE.0000000000000074

125. Boissonnault JS, Evans K, Tuttle N, Hetzel SJ, Boissonnault WG. Reliability of the ECHOWS Tool for assessment of patient interviewing skills. *Phys Ther*. 2016;96(4):443-455. doi:10.2522/ptj.20150172

126. Seif GA, Kraft SV, Bowden MG, Boissonnault JS. Intra-rater reliability of the ECHOWS Tool for real-time assessment of physical therapy student interviewing skills: A pilot study. *Health Prof Educ*. 2019;5(2):146-151. doi:10.1016/j.hpe.2018.05.001

127. Fisher KA, Shobeiri SA, Nihira MA. The use of standardized patient models for teaching the pelvic floor muscle examination. *J Pelvic Med Surg*. 2008;14(5):361-368. doi:10.1097/SPV.0b013e318130f579

128. Williams R, Sanford J, Stratford PW, Newman A. Grading written essays: A reliability study. *Phys Ther*. 1991;71(9):679-686.

129. Sattelmayer KM, Jagadamma KC, Sattelmayer F, Hilfiker R, Baer G. The assessment of procedural skills in physiotherapy education: A measurement study using the Rasch model. *Arch Physiother*. 2020;10:9. doi:10.1186/s40945-020-00080-0

130. Dorsey LL, Kelly PV, Luetkemeyer PB, Lojovich JM. Use of an academic professional behavior assessment and intervention to promote professional socialization. *J Allied Health*. 2018;47(3):210-216.

131. Edgeworth Ditwiler R, Lee Swisher L, Reddien Wagner B, Anderson SA. A model for professionalism evaluation: Using the RISE Assessment Tool across DPT didactic and integrated clinical education. *Internet J Allied Health Sci Pract*. 2023;22(1):1-16.

132. Nesbit KC, Fitzsimmons A. Grappling With professionalism: A developmental approach to a dynamic concept. *J Phys Ther Educ*. 2021;35(2):103-112. doi:10.1097/JTE.0000000000000174

133. Plack MM, Driscoll M, Blissett S, McKenna R, Plack TP. A method for assessing reflective journal writing. *J Allied Health*. 2005;34(4):199-208.

134. Pechak C, Dillon L, Umucu E. Improving patient-provider communication: Evolution of a tool to assess physical therapist students’ Spanish-language proficiency. *Health Commun*. 2019;34(12):1433-1440. doi:10.1080/10410236.2018.1495161

135. Riolo L. Reliability of assessing psychomotor tasks in physical therapy curricula. *J Phys Ther Educ Am Phys Ther Assoc Educ Sect*. 1997;11(1):36-39.

136. Emmert MC, Cai L. A pilot study to test the effectiveness of an innovative interprofessional education assessment strategy. *J Interprof Care*. 2015;29(5):451-456. doi:10.3109/13561820.2015.1025373

137. Zhang C, Miller C, Volkman K, Meza J, Jones K. Evaluation of the team performance observation tool with targeted behavioral markers in simulation-based interprofessional education. *J Interprof Care*. 2015;29(3):202-208. doi:10.3109/13561820.2014.982789

138. Tappan RS, Hedman LD, López-Rosado R, Roth HR. Checklist-Style Rubric Development for Practical Examination of Clinical Skills in Entry-Level Physical Therapist Education. *J Allied Health*. 2020;49(3):202-207.

139. Clark CR, Bialocerkowski A. Enhancing entry-level physiotherapy student learning in interpreting radiology -- An action research project. *Internet J Allied Health Sci Pract*. 2016;14(4):1-7.
